# FTIR–PCA Approach on Raw and Thermally Processed Chicken Lipids Stabilized by Nano-Encapsulation in β-Cyclodextrin

**DOI:** 10.3390/foods11223632

**Published:** 2022-11-14

**Authors:** Nicoleta Gabriela Hădărugă, Cosmina Andrea Chirilă, Raymond Nandy Szakal, Iulia Maria Gălan, Marius Daniel Simandi, Gabriel Stelian Bujancă, Ioan David, Adrian Riviş, Sorin Mihai Stanciu, Daniel Ioan Hădărugă

**Affiliations:** 1 Nicoleta Gabriela Hădărugă Timişoara, Calea Aradului 119, 300645 Timişoara, Romania; 2 Doctoral School “Engineering of Vegetable and Animal Resources”, University of Life Sciences “King Mihai I” from Timişoara, Calea Aradului 119, 300645 Timişoara, Romania; 3 Department of Economy and Company Financing, University of Life Sciences “King Mihai I” from Timişoara, Calea Aradului 119, 300645 Timişoara, Romania; 4 Department of Applied Chemistry, Organic and Natural Compounds Engineering, Polytechnic University of Timişoara, Carol Telbisz 6, 300001 Timişoara, Romania

**Keywords:** chicken lipids, cyclodextrins, nano-encapsulation, Fourier transform infrared spectroscopy, thermal analysis, principal component analysis, ATR-FTIR–PCA coupling technique

## Abstract

This study evaluated similarities/dissimilarities of raw and processed chicken breast and thigh lipids that were complexed by β-cyclodextrin, using a combined FTIR–PCA technique. Lipid fractions were analyzed as non-complexed and β-cyclodextrin-complexed samples via thermogravimetry, differential scanning calorimetry and ATR-FTIR. The lipid complexation reduced the water content to 7.67–8.33%, in comparison with the β-cyclodextrin hydrate (~14%). The stabilities of the complexes and β-cyclodextrin were almost the same. ATR-FTIR analysis revealed the presence of important bands that corresponded to the C=O groups (1743–1744 cm^−1^) in both the non-complexed and nano-encapsulated lipids. Furthermore, the bands that corresponded to the vibrations of double bonds corresponding to the natural/degraded (*cis*/*trans*) fatty acids in lipids appeared at 3008–3011 and 938–946 cm^−1^, respectively. The main FTIR bands that were involved in the discrimination of raw and processed chicken lipids, and of non-complexed and complexed lipids, were evaluated with PCA. The shifting of specific FTIR band wavenumbers had the highest influence, especially vibrations of the α(1→4) glucosidic bond in β-cyclodextrin for PC_1_, and CH_2/3_ groups from lipids for PC_2_. This first approach on β-cyclodextrin nano-encapsulation of chicken lipids revealed the possibility to stabilize poultry fatty components for further applications in various ingredients for the food industry.

## 1. Introduction

Poultry production in the European Union (EU) has continuously increased during recent years, with more than 25% growth in the last decade [1]. Poultry are especially grown for meat and eggs, with the important parts being broiler chickens and laying hens. The productivity of chicken meat was more than 11 × 10^6^ tons at the EU level in the year 2020. The highest productivity came from Poland, Spain, France, Italy and Germany [2]. Chickens (*Gallus gallus domesticus* L.) are grown in classical or organic farms, and the feed is the most important aspect in regard to the final meat composition. Intensive poultry production was strongly developed in the interwar period, especially for species selected for meat or egg production. Moreover, meat processing was also developed for the marketing of chicken carcasses [3].

In recent years, special attention has been given to the growing of broilers and laying hens, as well as to the food safety and security of their corresponding products, including “ready-to-eat” specialties. There are well-established requirements for broiler welfare with regards to the maximum stocking density for intensive rearing (Council Directive 2007/43/EC, the “Broiler Directive”). On the other hand, housing condition regulations for extensive indoor and free range chickens, as well as for organic farming chickens, are found in Commission Regulation (EC) No 543/2008 (Marketing Standards Regulation), Council Regulation (EC) No 834/2007 (Organic Regulation) and Commission Regulation (EC) No 889/2008 of 5 September 2008, which lay down detailed rules for the implementation of Council Regulation (EC) No 834/2007 on the organic production and labelling of organic products with regard to organic production, labelling and control [4,5,6,7,8]. Special attention was given to the bioactive compound-enriched feed formulations that include antioxidants such as vitamin E, or omega-3 based feed products (e.g., linseed oil) [9,10,11,12,13].

The fatty acid profile of chicken lipids depends on the feed composition and growing conditions used (conventional, organic or free range), the chicken parts and their ages [14,15,16,17,18,19,20]. Supplementation of feed with vegetable (e.g., linseed, flaxseed or hemp) or fish oils significantly increases the omega-3 polyunsaturated fatty acid content [10,11,21,22,23].

The fatty acids in lipids are mainly bound as triglycerides. However, a small fraction of free fatty acids occurs. They are more easily degraded than the corresponding glycerides [24,25]. On the other hand, polyunsaturated fatty acids and those having cis double bonds are more susceptible to oxidation. Volatile aldehydes and oxo-acids are formed during oxidation via a radical mechanism. They significantly reduce the nutritional and sensorial quality of fat-based foods [26,27]. Poultry lipids are rich in mono- and polyunsaturated fatty acid glycerides. Consequently, they are susceptible to degradation by oxidation. The presence of oxygen, transition metals (e.g., Fe and Cu) or enzymes, as well as heat and light, can accelerate lipid oxidation [25]. Lipid oxidation and microbial contamination affect the meat color, flavor and tenderness, and allow for evaluation of the shelf life of the meat [28]. Elevated temperature is another factor that enhances lipid degradation levels. Thus, the oleic acid content decreases from 37.7% to ~33% after conventional and pressure frying of chicken meat [29]. Moreover, the isomerization of natural unsaturated fatty acids to harmful *trans* isomers can occur at higher temperatures. The trans fatty acid content increases during the deep-frying process (temperature > 200 °C) of chicken fillets in palm oil, especially for 18:2 (9*c*, 12*t*) and 18:2 (9*t*, 12*c*) [30]. On the contrary, long-term storage (16 weeks at −16 °C or 24 weeks at −20 °C) and repeated freeze-thaw processing (up to four cycles) of the chicken breast, thigh, skin and liver do not significantly affect long-chain polyunsaturated fatty acid composition [31].

Extending the shelf life of poultry products can be performed using various techniques. They imply reduction of the lipid oxidation level and possible microbial contaminations. In this regard, packaging and antioxidant additions are often used. Vacuum packaging is a more appropriate method for the packaging of chicken breast meat in comparison with air or modified atmosphere packaging. However, the packaging method had only minor impact during refrigerated storage [32]. The addition of antioxidants such as α-tocopherol, α-tocopheryl acetate or ascorbic acid to the linseed- or sunflower-based feed, was found to protect the ground and vacuum-packaged raw or cooked dark chicken meat against oxidation during seven months of storage at −20 °C [33,34]. On the other hand, the pretreatment of chicken breast with black garlic extract was observed to provide significant protection against lipid oxidation; moreover, the addition of maltodextrin-encapsulated extract enhanced the antioxidant effect [35]. The effect of various spice and herb extracts (allspice, bay leaf, black seed, cardamom, caraway, clove, nutmeg, basil, garlic, onion, oregano, rosemary and thyme) on the fatty acid composition of the chicken meat during refrigerated storage was also demonstrated [36].

Nano-encapsulation is an appropriate technique for protecting the less stable compounds against oxidative degradation. It can be easily combined with the above-mentioned methods to increase lipid stability. Cyclodextrins (CDs) were proven to have protective effects for free fatty acids and their glycerides via molecular encapsulation of the geometrically compatible bioactive compounds, and reduction in the access of oxygen or air to the reaction sites [37,38]. CDs are cyclic oligosaccharides that have six (α-CD), seven (β-CD) or eight (γ-CD) α-d-glucopyranose units for the most known natural CDs. They have a highly hydrophilic exterior (high water solubility) and a hydrophobic cavity that can partially or totally accommodate hydrophobic molecules such as fatty acids and glycerides [39,40,41,42]. As a consequence, their apparent water solubility, oxidative stability and bioavailability, including controlled release, are increased [43]. There have been many studies on the nano-encapsulation of various fish oils, such as the common barbel (*Barbus barbus* L.), Pontic shad (*Alosa immaculata* Bennett), European wels catfish (*Silurus glanis* L.), common bleak (*Alburnus alburnus* L.), common nase (*Chondrostoma nasus* L.), European anchovy (*Engraulis encrasicolus* L.) and Atlantic salmon oil (*Salmo salar* L.), by natural or modified CDs [44,45,46,47]. CDs were also combined with other encapsulation matrices, such as polycaprolactone, gum arabic, sodium caseinate or whey protein concentrate, for fish oil encapsulation [48,49,50,51,52]. Animal fats are less studied for CD nano-encapsulation, in comparison with vegetable oils (e.g., common bean lipid fractions, soybean, kenaf seed, perilla or pumpkin oils) [53,54,55,56,57].

Chemometric tools are powerful techniques for classifying and evaluating the quality of food products. Among them, principal component analysis (PCA) allows for the identification of properties that are involved in the classification of the samples, as well as for the identification of their similarities/dissimilarities, and adulterations or degradations [58,59,60,61,62,63,64,65,66]. There are applications of PCA and other multivariate statistical techniques to the classification and evaluation of the quality and adulteration of vegetable and animal products [58,59,61,62,64,65,67,68,69,70,71,72].

Among animal products, milk and dairy products as well as animal fats have also been studied using the coupling of modern analyses and chemometrics. There are studies on the evaluation of milk and dairy products using multivariate statistical analysis techniques. A classification of bovine and buffalo anhydrous milk fat fractions and infant formula fats was performed using PCA applied on differential scanning calorimetry (DSC), FTIR and colorimetric data [73]. In another study, PCA was applied for the evaluation of the quality of milk powders subjected to fat replacement with different proportions of vegetable oils [74]. Vegetable fat can be easily detected in adulterated fresh cheeses using hierarchical cluster analysis (HCA) on the DSC data [63]. Among animal fats, a classification of pork back fat samples was performed using Raman spectroscopy and partial least squares (PLS) [75]. An interesting study was performed for the classification and quality evaluation of lipid fractions of Mangalitza (*Sus scrofa domesticus*), using gas chromatography–mass spectrometry coupled with PCA (GC–MS–PCA) [60]. However, there have been many studies on the application of vibrational spectroscopy and chemometrics for the evaluation of the adulteration and quality of edible oils and fats [66]. On the contrary, there are almost no studies on CDs and their animal fat complexes using chemometric tools.

In this study, an evaluation of the similarities/dissimilarities of the raw and processed chicken breast and thigh lipids complexed by β-CD was performed for the first time, using a combined attenuated total reflectance-Fourier transform infrared spectroscopy (ATR-FTIR)-PCA technique. It was expected that the protective capability of β-CD can be easily and rapid revealed by this vibrational spectroscopy.

## 2. Materials and Methods

### 2.1. Chicken Samples and Lipid Fraction Separation

Chicken samples (*Gallus gallus domesticus* L.) were purchased from the local market in 2019. According to the Romanian producer (Prăjeşti, Bacău, Romania; coordinates 46.6529° N and 26.9853° E, altitude 179 m), chickens were grown in conventional farms and fed with 70% corn. The nutritional values were as follows: fat 11.4%, carbohydrates 0.4%, proteins 20.6% and salt 0.18%. The energetic value was 781 kJ/100 g. Both the breast and thigh (with skin and bones) were immediately separated using a knife. Half of the samples were used for the manual separation of raw lipid fractions that were stored at 4 °C until analyses and β-CD complexation; the other half was cut into small pieces (~50 mm) and subjected to thermal processing by boiling. The boiling process was performed in a 6 L aluminum pressure cooker (Tefal Classic 6 L, Rumilly, Haute-Savoie, France) for 1 h at a meat:water ratio of 1:4 (mass to volume). After cooling, the liquid was separated, and the meat residue was pressed to recover as much liquid as possible. The combined liquid part was centrifuged for 15 min at 3200 rpm, using a Heraeus AG centrifuge (Hanau, Germany). The supernatant was stored at 4 °C for 12 h, and the semi-solid fat layer was separated, dried on absorbent cellulose paper, and stored again at 4 °C until further analyses and β-CD complexation. Unprocessed (raw) and thermally processed chicken breast and thigh lipid fractions were coded as “BU/BP” and “TU/TP”, respectively. Duplicate samples were used in this study (codes “BUa” and “BUb” for the raw breast lipid fractions, and “TUa” and “TUb” for the raw thigh lipid fractions; only single samples were used for the processed breast and thigh lipid fractions, codes “BPa” and “TPa”).

### 2.2. Materials and Reagents

The transesterification of the lipid glycerides to the more volatile fatty acid methyl esters (FAMEs) used methanol-boron trifluoride solution (20% BF_3_, Merck & Co., Inc., Rahway, NJ, USA), hexane (GC grade) and anhydrous sodium sulfate from Merck KGaA, Germany, as well as sodium chloride (reagent grade, Reactivul, Bucharest, Romania). Evaluation of the FA profile of the lipid fraction was performed using the FAME37 standard mixture, and the C_8_-C_20_ linear alkane standard mixture (both from Sigma-Aldrich, St. Louis, MO, USA). For the complexation of chicken lipid fractions, β-CD hydrate (Kleptose^®^, purity > 98%, α-CD max. 0.25%, γ-CD max. 0.25%, water content 14.0%, Roquette Frères S.A., Lestrem, France) and ethanol (96%, *v*/*v*, ChimReactiv, Bucharest, Romania) were used.

### 2.3. Gas Chromatography–Mass Spectrometry (GC–MS)

The FA profile of the raw and thermally processed lipid fractions was evaluated using GC–MS analysis of the derivatized samples [76,77]. The derivatization was performed via transesterification of triglycerides (the main class compounds in lipid fractions) and related compounds to the more volatile FAMEs. The derivatization took place in a 100 mL round-bottom flask using ~100 mg of the lipid sample, and 5 mL of BF_3_·MeOH solution. The mixture was refluxed for 30 min and cooled. Then, 5 mL of hexane was added, and the mixture was refluxed for another 30 min. After cooling, 15 mL of NaCl saturated solution was added and the mixture was vigorously stirred for 15 s. The mixture was completed with the NaCl solution for a better separation of the hexane layer that contained FAMEs. The organic layer was dried over anhydrous Na_2_SO_4_ for 24 h in the dark, and the samples were stored at 4 °C until analysis. Among FAMEs, acetals of the aldehydes, as well as acetals of the ω-formylated carboxylic acid methyl esters could also be formed by derivatization of the corresponding compounds that resulted during possible lipid degradation [60].

GC–MS analysis of the derivatized lipid samples was performed using a GC Hewlett Packard 6890 Series, coupled with a Hewlett Packard 5973 Mass Selective Detector. The GC column was a Zebron 5-MS with a length of 30 m, 0.25 mm inner diameter and 0.25 μm film thickness. The temperature program used was from 50 to 300 °C, with a heating rate of 6 °C/min, and injector and detector temperatures of 300 °C. Helium was selected as the carrier gas, and the sample injection volume was 2 μL. In order to protect the GC to MS line system, a solvent delay of 4 min was set. The MS energy source had an EI of 70 eV and a temperature of 150 °C. The MS scan range was set to 50–300 atomic mass units (amu) and a scanning rate of 1 s^−1^.

Two methods were applied for identifying the compounds. The first method was based on the retention indices (*RI*) that were determined using the FAME37 standard mixture, the C_8_-C_20_ linear alkane standard solution and the retention times (*RT*) for the separated compounds. The best *RI* versus *RT* correlational equation had a fourth-order parabolic form (1) [77]:

*RI* = 672.792 + 73.268·*RT* − 3.287·*RT^2^* + 0.148·*RT^3^* − 0.00201·*RT^4^* (1)

The second method used the MS spectra of the separated compounds, which were compared with those from the NIST/EPA/NIH Mass Spectral Library 2.0 (2011), using NIST MS Search 2.0 software. The best matching probability allowed for identification of the compound, and the result was compared with that obtained by the first method.

### 2.4. Preparation of the β-Cyclodextrin/Lipid Complexes

Kneading was the most appropriate technique for obtaining the β-CD/lipid complexes. The β-CD:lipid ratio was determined according to the GC–MS results for lipid fractions, taking into account the main FAs in these samples. Thus, the mean molar mass for the lipid fractions was 861 g/mol (considering triglycerides as the main constituents, and a purity of 90.1%; other non-volatile constituents such as cholesterol and phospholipids were present in the chicken lipids [77,78,79]). On the other hand, β-CD had a water content of 14%, and this information was considered for the determination of the β-CD:lipid molar ratio. Approximately 1 mmol of β-CD (1.30–1.32 g of β-CD hydrate) was dissolved in 2 mL of distilled water in a pre-heated mortar (55 °C). The lipid fraction (0.91–0.98 g corresponding to ~1 mmol) was added to the β-CD solution as an emulsion in ethanol. The mixture was kneaded for at least 45 min until a pasty consistency was obtained. The wet complex was dried in the dark for 24 h at room temperature. The dried complex was then milled into a powder and stored at 4 °C until analysis. Complexes were obtained as duplicate samples for the β-CD/raw lipid complexes (codes “XBUa”, “XBUb”, “XTUa” and “XTUb” for the raw breast and thigh cases, respectively). The β-CD/processed lipid complexes were obtained as single samples (codes “XBPa” and “XTPa” for the processed breast and thigh cases, respectively).

### 2.5. Attenuated Total Reflectance-Fourier Transform Infrared Spectroscopy (ATR-FTIR)

ATR-FTIR analyses of the chicken lipids and the corresponding β-CD complexes were performed on an FTIR Bruker Vertex 70 (Bruker Optik GmbH, Ettlingen, Germany) equipped with a single-reflection Platinum diamond ATR. The DLaTGS detector had a spectrum range of 12,000–250 cm^−1^ and a sensibility of D* > 2108 cm·Hz^1/2^·W^−1^. The ATR-FTIR acquisition range for the samples was 4000–400 cm^−1^, with a resolution of 4 cm^−1^ and 128 scans/sample. ATR-FTIR determinations were made in air, using sample masses of 10–40 mg. The acquisition and handling of the ATR-FTIR data were performed using OPUS ver. 7.2 software (Bruker Optik GmbH 2012, Ettlingen, Germany). FTIR analyses were performed in triplicate for every individual sample (raw and processed lipid fractions, β-CD hydrate and β-CD/lipid complexes), and the mean values of the FTIR band wavenumbers and intensities were used for the PCA analysis.

### 2.6. Thermogravimetry–Differential Thermogravimetry (TG–DTG)

TG–DTG analysis of the chicken lipids and the β-CD/lipid complexes was performed for the evaluation of the thermal stability of the samples during heating. A Netzsch TG 209F1 Libra equipment (Netzsch Group, Selb, Germany) with a temperature program of 25–500 °C and a heating rate of 10 °C/min was used. Nitrogen was used for both purge and protection, with a flow of 40 mL/min. The acquisition and handling of the TG–DTG data were performed with Netzsch Proteus-Thermal Analysis ver. 6.1 software from the same producer.

### 2.7. Differential Scanning Calorimetry (DSC)

A Netzsch 204 F1 Phoenix (Netzsch Group, Selb, Germany) apparatus was used for the DSC analysis of the above-mentioned samples. The analysis conditions were the same as those used for TG (temperature range and rate of 25–500 °C and 10 °C/min, nitrogen purge and protective flow of 40 mL/min), as well as the software used for the acquisition and handling of the DSC data.

### 2.8. Principal Component Analysis (PCA)

PCA is a powerful multivariate statistical analysis technique that allows for discriminating objects only on the basis of independent variables. Unscrambler 6.0 (Camo, Trondheim) was used for evaluating the similarity/dissimilarity of the chicken lipids and β-CD/lipid complexes, or the impact of various ATR-FTIR variables to these classifications. ATR-FTIR band wavenumbers and intensities were used (separately or combined) as independent variables for the PCA analysis. Raw (unprocessed) and processed chicken lipid fractions (breast and thigh), as well as the corresponding β-CD/unprocessed chicken lipid and β-CD/processed chicken lipid complexes, were considered as samples in the PCA analysis. The PCA analysis was performed using the centered ATR-FTIR data and the cross-validation method.

## 3. Results and Discussion

### 3.1. Analysis of the Raw and Processed Chicken Lipids

#### 3.1.1. Thermal Analyses of the Chicken Lipids

**Figure 1:**
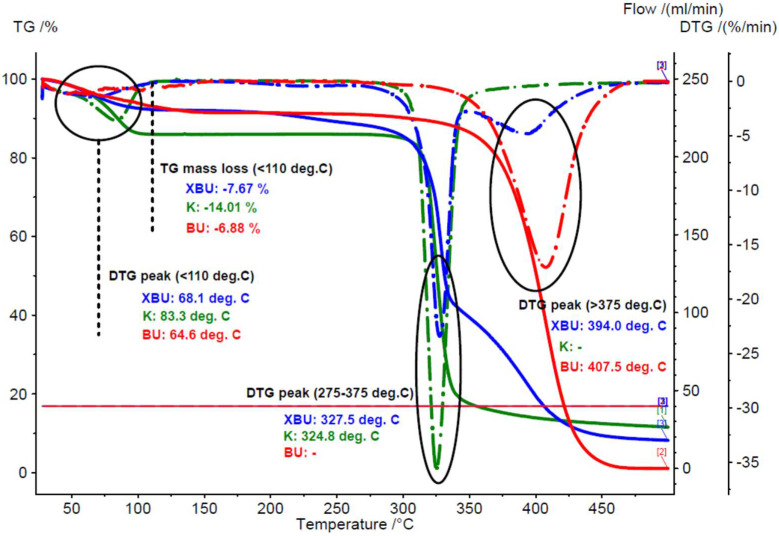
Superimposed TG–DTG thermograms for the β-cyclodextrin/raw breast chicken lipid complex (blue), β-cyclodextrin hydrate (green), and raw breast chicken lipid fraction (red) (codes “XBU”, “K” and “BU”, duplicate samples “a”).

**Figure 2:**
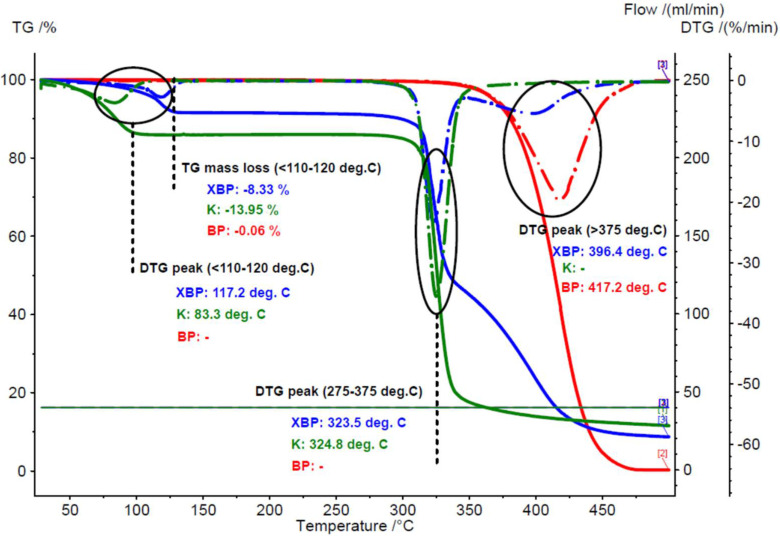
Superimposed TG–DTG thermograms for the β-cyclodextrin/processed (boiling) breast chicken lipid complex (blue), β-cyclodextrin hydrate (green), and processed (boiling) breast chicken lipid fraction (red) (codes “XBP”, “K” and “BP”, duplicate samples “a”).

Chicken lipids were analyzed via thermal, spectroscopic and chromatographic methods. Thermal methods allow for evaluation of the thermal and oxidative stability of the samples by determining the mass loss and/or the calorimetric effects during heating. TG analysis of the raw chicken breast and thigh lipids reveals a mass loss up to 110 °C that corresponds to water or moisture dissociation from the lipid matrix, as well as an important mass loss at higher temperatures > 275 °C, corresponding to the degradation of the lipid components. The moisture content of the raw lipid fractions was in the range of 4.5–7.0%, slightly lower for the breast cases (*RSD*~23%). A mass loss of 2.21 and 4.03% was also observed for the raw breast and thigh lipids in the range of 110–275 °C, with an important mass change up to 150 °C (see Figures S1 and S2 in the Supplementary Materials file). It must be noted that the raw samples were less homogenous in comparison with the processed ones. For the processed lipid samples, the moisture content was much lower. Only a mass loss of 0.06 and 0.31% was observed for the breast and thigh lipid samples that were obtained by the boiling-pressing method up to 110 °C. A mass loss in the range of 110–275 °C was also less significant (0.21 and 0.28%, respectively). The degradation behavior of the samples was completely different for the raw and processed lipid fractions. The degradation began at ~275 °C, but the major mass loss appeared after 350 °C. The mass changes for the raw breast and thigh lipid samples were 89.34 and 87.73%, respectively, while for the corresponding processed samples these values were >99% (*RSD* < 1.2%). Moreover, the maximum degradation rate for the raw breast and thigh lipid samples appeared at lower temperature values of 409 and 413.7 °C, respectively, in comparison with the processed samples (417.2 and 415.3 °C, respectively), as was determined via DTG. All of the TG–DTG results are presented in Figure 1 and Figure 2, as well as in Figures S1–S3 in the Supplementary Materials file. This thermal behavior can be explained by the partial degradation of the lipid components during the boiling of the chicken meat, and the formation of some volatile or hydrophilic degradation compounds. Such compounds (aldehydes, formylated carboxylic acids, or dicarboxylic acids) were even lost by evaporation, or were water solubilized during processing [47,77,80]. Consequently, only more stable compounds (especially triglycerides) remained in the processed lipid samples.

**Figure 3:**
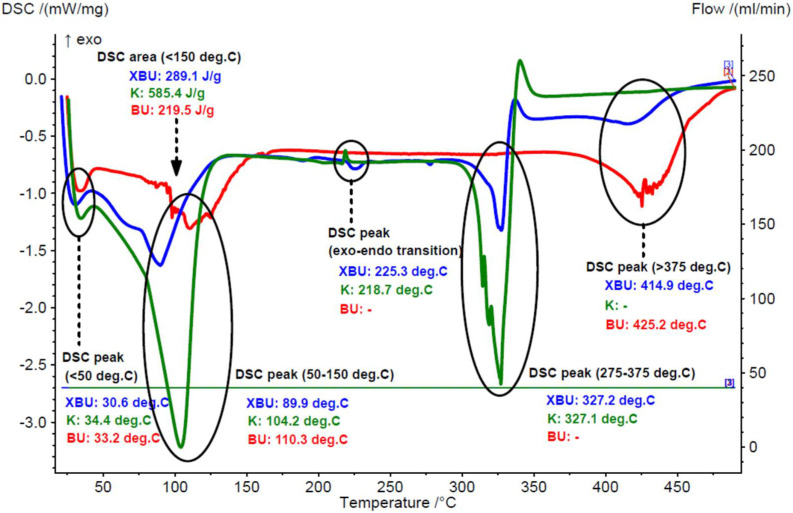
Superimposed DSC curves for the β-cyclodextrin/raw breast chicken lipid complex (blue), β-cyclodextrin hydrate (green), and raw breast chicken lipid fraction (red) (codes “XBU”, “K” and “BU”, duplicate samples “a”).

**Figure 4:**
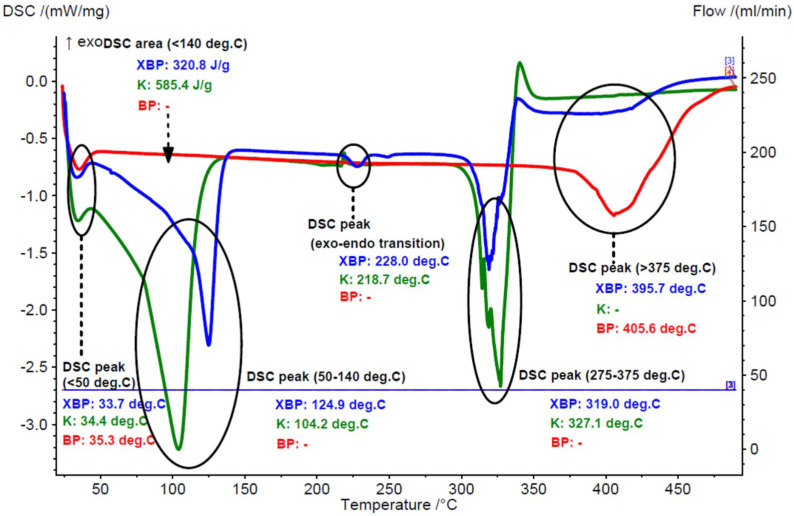
Superimposed DSC curves for the β-cyclodextrin/processed (boiling) breast chicken lipid complex (blue), β-cyclodextrin hydrate (green), and processed (boiling) breast chicken lipid fraction (red) (codes “XBP”, “K” and “BP”, duplicate samples “a”).

The DSC analysis results were in agreement with the TG–DTG data for the raw and processed chicken breast and thigh lipids. The calorimetric effect for the moisture dissociation was significantly higher for the raw samples (averages of 305.8 and 252.5 J/g for the raw breast and thigh lipids, respectively), in comparison with the processed samples (6.9–7.4 J/g). On the other hand, the maximum moisture dissociation effect appeared at similar temperatures in the range of 33.2–43.4 °C and 99.1–110.3 °C, corresponding to the surface water and strongly retained water molecules (or other volatiles), respectively. Degradation of the lipid components appeared at temperatures > 350 °C, with calorimetric effects of 185.4–273.4 J/g and maximum degradation temperatures at 405.6–424.6 °C (see Figure 3 and Figure 4, as well as Figures S4–S6 in the Supplementary Materials file).

#### 3.1.2. Fourier Transform Infrared Spectroscopy (FTIR) Analysis of the Chicken Lipids

**Figure 5:**
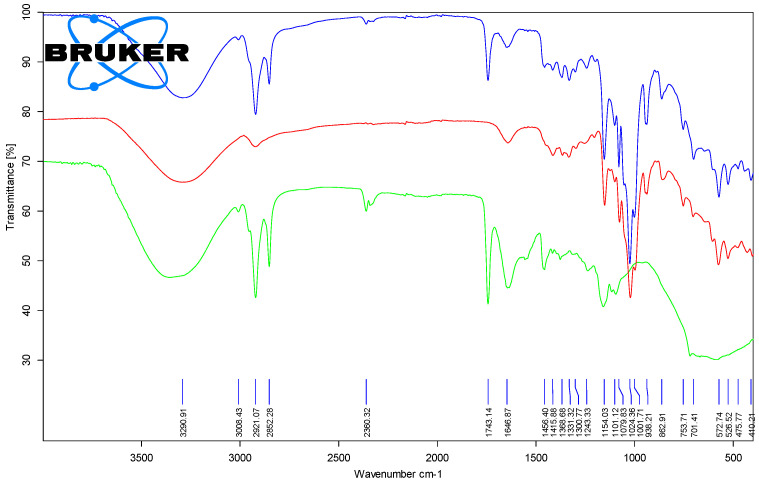
Superimposed ATR-FTIR spectra for the β-cyclodextrin/unprocessed chicken breast lipid complex (top—blue), β-cyclodextrin (middle—red) and lipid fraction from the unprocessed chicken breast (bottom—green) (duplicate samples “a”).

**Figure 6:**
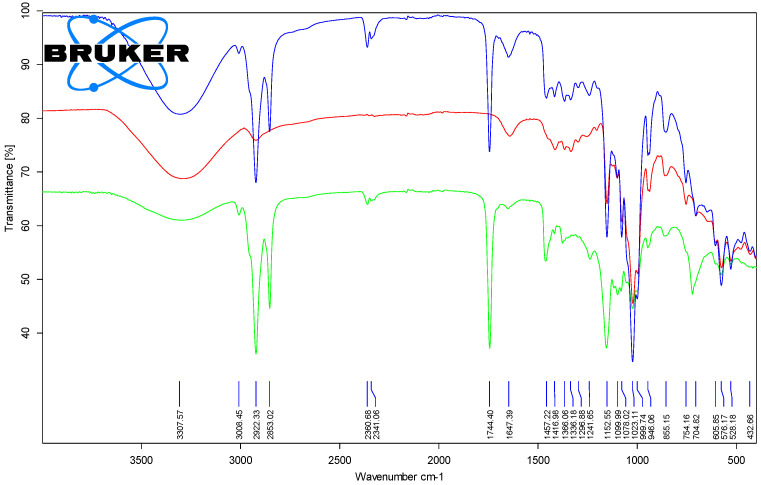
Superimposed ATR-FTIR spectra for the β-cyclodextrin/processed chicken breast lipid complex (top—blue), β-cyclodextrin (middle—red) and lipid fraction from the processed chicken breast (bottom—green) (duplicate samples “a”).

The most important FTIR band for the lipid fractions was the one that corresponded to the stretching vibration of the ester group in the triglycerides. It appeared at 1741.2–1743.5 cm^−1^, with differences that were not significant between the raw and processed samples. On the contrary, the stretching vibration of the cis R_1_HC=CHR_2_ groups in natural unsaturated fatty acid moieties appeared at higher wavenumbers for the processed lipid samples of 1649.8–1652.1 cm^−1^ in comparison with the raw samples (1634.6–1645.1 cm^−1^). A similar behavior was observed for the rocking of =C-H bending of the *cis* groups (1412.4–1416.8 and 1417.1–1417.5 cm^−1^ for the raw and processed samples). The FTIR band that corresponded to the bending vibration of the *trans* R_3_HC=CHR_4_ groups only appeared in the processed lipid samples at 946.0–946.7 cm^−1^, as a result of partial thermal degradation/isomerization of the natural fatty acid moieties. Other common FTIR bands for the lipid components were the symmetric and asymmetric stretching vibrations of the CH groups (3007.9–3009.1, 2952.8–2957.4, 2917.2–2921.9, and 2850.4–2852.7 cm^−1^), bending vibrations of the CH_2_ and CH_3_ groups (1455.7–1463.0, 1376.5–1379.4, 1237.4–1241.6 and 1154.4–1162.9 cm^−1^), C-O stretching vibrations at 1079.9–1098.7, 1052.7–1054.6 and 1022.3–1034.6 cm^−1^, as well as C-H out-of-plane deformation at 717.1–721.3 cm^−1^ (see Table 1, Figure 5 and Figure 6, as well as Figures S7 and S8 in the Supplementary Materials file).

The FTIR results were in accordance with the fatty acid profile that was determined via GC–MS after derivatization to the corresponding FAMEs, and was discussed in another study [77]. The monounsaturated fatty acids (MUFAs) were the most concentrated in the raw samples (29.6 and 26.4%, for the breast and thigh lipids, respectively), with oleic acid (as methyl ester) at a relative concentration of 21.5 and 19.7% (RI of 2113). Polyunsaturated fatty acids (PUFAs) had a lower concentration of 16.7 and 14.6% in the raw samples, the main compound being linoleic acid (RI of 2103). These results are in agreement with those of other studies on the fatty acid profile of chicken lipids, where oleic acid is the most concentrated [14,18,19,20]. On the other hand, the chickens used in this study were grown with corn, which have oleic and linoleic acids as their main fatty acids in the corn lipid fraction. Saturated fatty acids (SFAs) were in similar contents both in the breast and thigh lipid samples (23.1–23.7%), and had palmitic acid as the main compound (17.6–17.8%, RI of 1940). Processing of the lipid samples increased the SFA content by 2–5%, due to the higher thermal stability of such compounds. However, the other fatty acid classes had higher relative concentrations in the processed samples. This is probably due to changes in the overall hydrophobic compound types (including others than triglycerides) after boiling. This difference was revealed by the relative concentrations of the unidentified compounds (most probably non-triglycerides, which could not remain in the processed samples, or were degraded during boiling) in the processed lipids, in comparison with those from the raw samples. As a consequence, the unidentified compounds in the raw lipids were 28.8–35.3%, and only 16.8–23.0% in the processed lipids [77]. Furthermore, degradation/isomerization to the *trans* compounds was revealed after processing, especially for thigh samples, where *trans* elaidic acid was found at a higher content of 1.4%. Another degradation compound was hexanal (as dimethyl acetal after derivatization), which was 2–3 times more concentrated in the processed samples.

Protection of these labile hydrophobic compounds against degradation can be achieved using food-grade CD nano-encapsulation, to obtain formulations with enhanced properties (higher stability, controlled release and increased “apparent” water solubility and bioavailability), and in applications for various food ingredients and products.

### 3.2. Preparation and Analysis of the β-Cyclodextrin/Chicken Lipid Complexes

#### 3.2.1. Preparation of the β-Cyclodextrin/Chicken Lipid Complexes by Kneading

According to the literature, the kneading method is the most appropriate CD technique for nano-encapsulation of chicken lipid components [38,43,81]. The β-CD hydrate has a water content of ~14%. During the molecular encapsulation process, the fatty acid moieties of the triglycerides contained by chicken lipids progressively replace a part of the water molecules from the β-CD hydrate. Consequently, the water content of a β-CD/hydrophobic compound complex is lower [41,42,44,46,47,53]. According to this study, the recovering yield was higher for the β-CD/processed chicken lipid complexes in comparison with that in the raw samples (79.57 (±5.33) and 66.68 (±9.18)%, respectively). A higher recovery yield for the β-CD complexation of the processed chicken lipids was obtained, due to the more homogenous hydrophobic components that better interacted with the hydrophobic inner cavity of the β-CD [38].

#### 3.2.2. Thermal Analyses of the β-Cyclodextrin/Chicken Lipid Complexes

The above-mentioned aspects were also revealed by the thermal analyses of β-CD hydrate and its chicken lipid complexes. TG–DTG analysis of the β-CD hydrate revealed a water loss of 13.4 (±0.88)% up to 110 °C (maximum dissociation rate at 82.6 (±0.99)%), and almost no change up to the initiation of the degradation process (>275 °C, mass loss of 74.61 (±0.40)% and maximum degradation rate at 323.15 (±2.33) °C). On the other hand, β-CD/chicken lipid complexes exhibited different TG–DTG behavior. The water content of both β-CD/raw and processed lipid complexes was significantly lower after molecular inclusion of lipid triglycerides into the CD cavity (6.3–8.3%). An interesting difference between the maximum water dissociation rates for the β-CD/raw and processed lipid complexes was observed. Thus, this DTG parameter had similar values for the β-CD/raw lipid complexes, such as for β-CD hydrate (68.1–83.4 and 82.6 °C, respectively). On the contrary, the β-CD/processed lipid complexes had significantly higher values for the temperature corresponding to the maximum water dissociation rate (107.8–117.2 °C). This behavior was probably due to the dissociation of the strongly retained water and solvent (from the complexation process) molecules from the β-CD/lipid complexes [42]. Another difference appeared for the mass loss during the temperature range of 110–275 °C. It was consistently higher for the β-CD/chicken lipid complexes, in comparison with β-CD hydrate (0.6–4.1%, and only ~0.05% for β-CD hydrate). Regarding the degradation of the complexes, there were two DTG peaks that corresponded to β-CD degradation at 323.5–327.5 °C and lipid components degradation, after their dissociation from the early degraded β-CD (394.0–402.6 °C). The total mass change during degradations was in the range of 79.9–82.4%. Comparative TG–DTG plots for the β-CD/chicken lipid complexes, as well as for β-CD hydrate and non-complexed raw and processed lipid fractions, are presented in Figure 1 and Figure 2, respectively (for other thermograms, see Figures S9–S15 in the Supplementary Materials file).

According to the DSC analysis, the endothermal calorimetric effect of the water dissociation was significantly lower in the β-CD/chicken lipid complexes, in comparison with that for the β-CD hydrate. Thus, this calorimetric effect for complexes was in the range of 251.4–320.8 J/g, and slightly higher for the β-CD/processed lipid complexes. On the contrary, this effect was consistently higher for the β-CD hydrate (430.9–585.4 J/g). Moreover, the maximum dissociation rate was observed at higher temperatures for the β-CD/processed lipid complex (124.9 °C). Both the surface and strongly retained water dissociation calorimetric effects were in good agreement with the TG–DTG analysis. On the other hand, the degradation of β-CD hydrate and β-CD from the complexes showed a maximum rate in a narrow temperature range of 319.0–328.7 °C, while the degradation of lipid components was in the range of 372.9–414.9 °C. It was also observed through DSC analysis (not in the TG–DTG) that there was an endo-exothermic effect in the range of 225.3–228.8 °C for complexes, and ~218 °C for β-CD hydrate. This was due to the crystalline-amorphous transition effect in both β-CD and its complexes [41,42]. Comparative DSC curves that correspond to β-CD/chicken lipid complexes, β-CD hydrate and the raw or processed chicken lipid fractions, are presented in Figure 3 and Figure 4, as well as in Figures S16–S21 in the Supplementary Materials file.

#### 3.2.3. Fourier Transform Infrared Spectroscopy (FTIR) Analysis of the β-Cyclodextrin/Chicken Lipid Complexes

The presence of lipid components in the β-CD complexes was revealed by ATR-FTIR analysis. FTIR analysis of the β-CD/lipid complexes confirmed the presence of lipid components in the complexes through the existence of the characteristic absorption bands for the stretching vibrations of the ester groups in triglycerides at 1743.1–1744.4 cm^−1^ (Figure 5 and Figure 6, as well as Figures S22 and S23 in the Supplementary Materials file). This finding confirmed the modification of the band wavenumbers and intensities as a result of host–guest molecular encapsulation [45,46]. However, it is difficult to compare the absolute values from the FTIR data for the β-CD/chicken lipid complexes and the original components, since most of the FTIR bands appeared in both β-CD and lipids. What differentiated best was the vibration band that corresponded to the C=O carbonyl bond in the esters, which appeared in the triglycerides, but also in the bands that corresponded to the vibrations of =CH or C=C bonds (*cis* or *trans*) associated with the fatty acid residues in lipids. They also appeared in complexes at 3007.8–3011.0 cm^−1^, 1644.4–1650.1 cm^−1^, 1415.9–1418.0 cm^−1^ and 938.2–946.1 cm^−1^ for the corresponding vibrations (Table 1).

By comparing the FTIR spectra for the β-CD hydrate and the corresponding complexes with lipids from the chicken breast and thigh, almost all bands of the β-CD were also found in the triglycerides. However, their intensities differentiated these sample types. For example, the band that corresponded to the stretching and/or bending vibrations of the O-H, CH/CH_2_, C-C, C-O groups appeared in both components, with the complexes presenting bands that were slightly modified in terms of wavenumber and intensity (3290.4–3309.2 cm^−1^ for the stretching vibration of the O-H groups, 1078.0–1080.2 cm^−1^ for the stretching vibration of the C-C bonds, higher compared to that of β-CD of 1076.9–1077.0 cm^−1^, as well as 1051.4–1054.0 cm^−1^ for the stretching vibration of the C-O bonds, and 1415.9–1418.0 cm^−1^ for the in-plane bending vibration of the C-O bonds, Table 2). There were also some differences observed between the FTIR bands of the β-CD and its complexes, such as the stretching vibrations of the glucosidic groups, δ_C-O-C_, at 1152.6–1154.4 cm^−1^, or the stretching vibrations of the α(1→4) glucosidic ring at 938.2–946.1 cm^−1^ and 854.8–863.0 cm^−1^, corresponding to the CD rings, which however, overlapped with some bands of the lipid components. Thus, the presence of such FTIR bands in the case of complexes supported the presence of triglycerides in the β-CD complexes, but without allowing for evaluation of their means of interaction.

### 3.3. Principal Component Analysis (PCA) on the β-Cyclodextrin/Chicken Lipid Complexes

**Figure 7:**
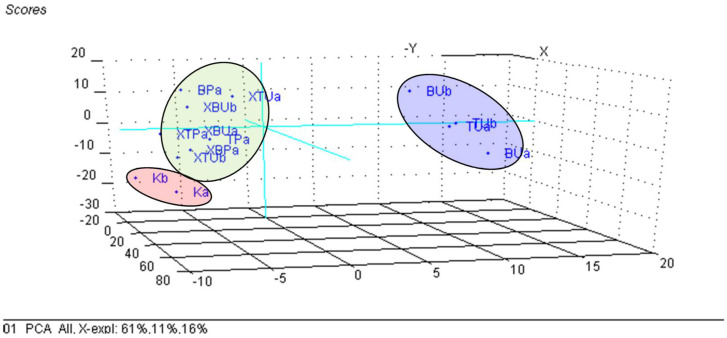
PC_3_ versus PC_1_, PC_2_ scores plot from the PCA analysis of all FTIR data (both intensities and wavenumbers) of the β-cyclodextrin/unprocessed and processed chicken breast and thigh lipid complexes (codes “XBU”, “XBP”, “XTU” and “XTP”), in comparison with β-cyclodextrin (code “K”) and non-complexed lipid fractions (codes “BU”, “BP”, “TU” and “TP”); “a” and “b” stand for duplicate samples.

**Figure 8:**
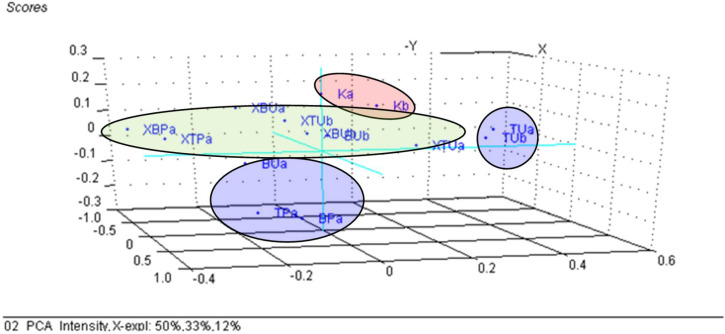
PC_3_ versus PC_1_, PC_2_ scores plot from the PCA analysis of the band intensities from the FTIR data of the β-cyclodextrin/unprocessed and processed chicken breast and thigh lipid complexes (codes “XBU”, “XBP”, “XTU” and “XTP”), in comparison with β-cyclodextrin (code “K”) and non-complexed lipid fractions (codes “BU”, “BP”, “TU” and “TP”); “a” and “b” stand for duplicate samples.

The changes in the wavenumbers and intensities of the ATR-FTIR bands of the β-CD/chicken lipid complexes, in comparison with the starting β-CD hydrate or the raw and processed lipid fractions, were difficult to evaluate. The multivariate statistical technique, PCA, allows for extraction of useful information from the large FTIR dataset. PCA processes the dataset by searching the direction through the independent properties space, in order to obtain a maximum variance. This direction is called the first principal component (PC_1_ or Factor 1), and retains the main information about the samples. Other PCs can be obtained in a similar way, but with restrictions of the orthogonality between them. As a result, the original axis system is converted into another one having PCs as the new axes. The translation coordinates provide the “Scores” plot that reveals the similarity/dissimilarity between the samples. On the other hand, the rotation coordinates provide the “Loadings” plot, which reveals the importance of the independent variables to the classification. Only a few PCs (generally two or three, e.g., PC_1_, PC_2_ and PC_3_) are sufficient for classification, according to the behavior of the residual variance plot. As a consequence, PCA significantly reduces the number of axes that retain the main information from the dataset [67,68,69,70]. Coupling of PCA with FTIR analysis for the β-CD/lipid complexes indicated an obvious classification of unprocessed lipid samples (codes “BU” and “TU”) on the right side of the PC_3_ versus PC_1_, PC_2_ score plot (Figure 7), while the samples obtained by thermal processing (codes “BP” and “TP”) were located on the left side. In addition, β-CD (coded as “K”) clustered at the bottom of this graph, and β-CD/chicken lipid complexes (codes “XBU”, “XTU”, “XBP” and “XTP”) are located in the central part, with a sub-grouping according to the level of processing [47,77]. The most important variables for this classification were the stretching vibrations of the O-H groups and the α(1→4) glucosidic bonds in cyclodextrins for PC_1_, on the positive and negative directions, respectively; the bending vibrations for the CH/CH_2_ or COC bonds on the positive side, respectively; and bending of the OH group and the stretching of the *cis* R_1_CH=CHR_2_ groups for the negative part of the PC_2_ component. The stretching vibration of the CO bonds in the lipids was important for the classification of the samples along the PC_3_ axis (see Figures S24 and S25 in the Supplementary Materials file). The intensity of the FTIR bands seems to be less important for the classification of the samples; only the processed lipids were located in the lower part of the score plot, while β-CD hydrate samples were grouped in the upper part, with the complexes in the center. The explained variances for the first three PCs were 50, 33 and 12%, respectively (Figure 8 and Figure S26 in the Supplementary Materials file).

For the results of the PCA–FTIR analyses for the β-CD/unprocessed and processed chicken breast and thigh lipid complexes, respectively, the raw materials used in the complexation process were completely different, if only the wavenumbers of all of the characteristic FTIR bands were used as input variables. The raw lipid samples are clearly differentiated on the right side of the PC_3_ versus PC_1_, PC_2_ scores plot, while all of the other samples are located in the central-bottom part of this plot (Figure S27 in the Supplementary Materials file). In addition, the samples in this area were sub-classified as β-CD (“K”) in the lower part, responsible for the classification being also the stretching vibrations that corresponded to the OH or α(1→4) glycosidic bonds and the bending vibrations of the CH/CH_2_ and OH groups, respectively, or the stretching vibrations of the cis C=C and CO bonds (Figure S28 in the Supplementary Materials file).

**Figure 9:**
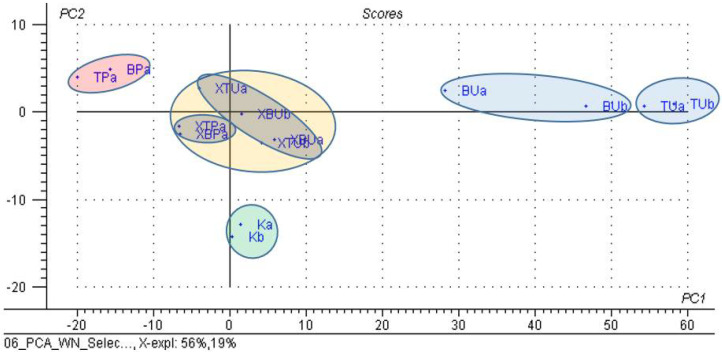
PC_2_ versus PC_1_ scores plot from the PCA analysis of the significant band wavenumbers from the FTIR data of the β-cyclodextrin/unprocessed and processed chicken breast and thigh lipid complexes (codes “XBU”, “XBP”, “XTU” and “XTP”), in comparison with β-cyclodextrin (code “K”) and non-complexed lipid fractions (codes “BU”, “BP”, “TU” and “TP”); “a” and “b” stand for duplicate samples.

**Figure 10:**
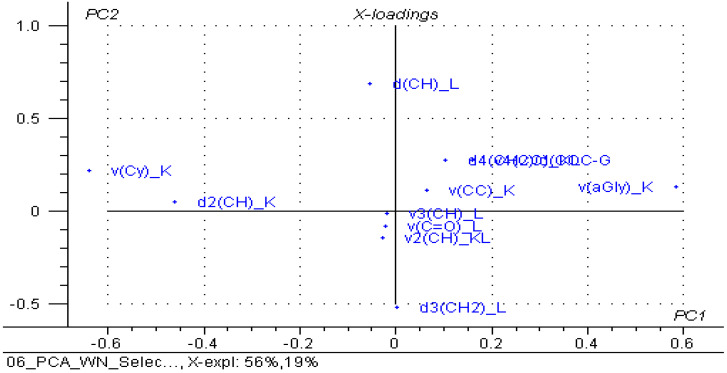
PC_2_ versus PC_1_ loadings plot from the PCA analysis of the selected FTIR band wavenumber data (see the text) of the β-cyclodextrin/unprocessed and processed chicken breast and thigh lipid complexes, β-cyclodextrin and non-complexed lipid fractions; variable codes contain “v” or “d” for the stretching and bending vibrations of the corresponding bonds (in parenthesis), followed by the source (“K” for β-cyclodextrin and “L” for lipids); e.g., “v(aGly)_K” stands for the variable corresponding to the wavenumber of the stretching vibration of the α(1→4) glucosidic bond from β-CD at ~859 cm^−1^ (for all variable codes, see Table S1 from the Supplementary Materials file).

When selecting variables, only the significant wavenumbers from the FTIR analysis (see Table S1 from the Supplementary Materials file) for β-CD/unprocessed or thermally processed chicken breast and thigh lipid complexes (codes “XBU”, “XBP”, “XTU” and “XTP”), respectively, of the β-CD hydrate (code “K”), and the corresponding non-complexed lipid fractions (codes “BU”, “BP”, “TU” and “TP”), the classification was much improved. A grouping of the lipid fractions was observed on the right side of the PC_2_ versus PC_1_ scores plot, but also their sub-classification, especially for unprocessed lipids; the processed lipid samples are located on the left side of the graph, and the starting β-CD samples are located on the bottom. All of the β-CD/chicken lipid complexes are centrally located, but again, a sub-classification occurs for complexes with processed lipids in the center-left zone; meanwhile, all of the other complexes with unprocessed lipids were sub-classified in the center-right zone (Figure 9). This clustering was also observed in the PC_3_ versus PC_1_, PC_2_ scores plot (Figure S29 from the Supplementary Materials file) for the β-CD on the left side, while the non-complexed lipids on the right side, respectively, with the complexes in the central area. The important variables for PC_1_ were, in particular, the wavenumbers that corresponded to the stretching vibrations for the glucosidic ring of the β-CD, and the bending vibrations for CH and CH_2_ groups in the lipids for the PC_2_, respectively (Figure 10). The explained variance in the data is represented by the first three PCs in a proportion of 86% (56% for PC_1_, 19% for PC_2_ and 11% for PC_3_), these PCs being sufficient for the discrimination of β-CD/lipid complexes from the raw or processed chicken lipid fractions, with β-CD hydrate used in the molecular nano-encapsulation (Figure S30 from the Supplementary Materials file).

## 4. Conclusions

Chicken lipid nano-encapsulation using CDs is the first approach that addresses the thermal and oxidative stabilizing technique of such food materials. The relatively higher content of unsaturated fatty acid glycerides in the breast and thigh lipid fractions reduced the stability of these food materials. Their nano-encapsulation using food-grade natural and cheap β-CD was performed by the kneading method, with recovery yields of up to 80%. Almost no changes (excepting the water or moisture release) on the β-CD/chicken lipid sample mass were observed up to 220 °C, which is significantly higher that the frying temperature for such food products. The presence of the chicken lipid triglycerides in the β-CD complexes was proven with ATR-FTIR, which revealed shifting of some stretching and bending bands of both starting materials. The coupling of this spectroscopic method with multivariate analysis techniques allowed for discriminating between raw and processed chicken lipids, even non-complexed or β-CD nano-encapsulated. Moreover, the characteristic bands of the β-CD discriminate the level of lipid processing, while the specific bands for lipids discriminate the level of β-CD complexation. This first study on the CD nano-encapsulation of chicken lipids can be continued by applying such materials into various food products, and evaluating the stability/degradation of lipid components during industrial processing and “at home” preparation with various methods (boiling, frying, baking, etc.).

The overall conclusion of the study is that food-grade CD nano-encapsulation of chicken lipids can provide innovative materials with improved thermal and oxidative stability for further application as ingredients in the food industry.

## Figures and Tables

**Table 1:** Comparisons of the ATR-FTIR band assignments of the lipid fractions (chicken breast and thigh, unprocessed and processed) and the corresponding β-cyclodextrin/lipid complexes.

Band Assignments for the Lipid Fractions (cm^−1^)	Band Assignments for the β-CD/Lipid Complexes (cm^−1^)	Description
3287.4–3357.8	3290.4–3309.2	ν_O-H_, stretching vibration
3007.9–3009.1	3007.8–3011.0	ν^s^_=CH_, symmetric stretching vibration
2952.8–2957.4	2951.8–2954.0	ν^as^_C-H_, asymmetric stretching vibration
2917.2–2921.9	2921.1–2922.7	ν^as^_C-H_, asymmetric stretching vibration
2850.4–2852.7	2852.3–2853.5	ν^as^_CH_, asymmetric stretching vibration
1741.2–1743.5	1743.1–1744.4	ν_C=O_, stretching vibration of the ester groups from triglycerides
1634.6–1652.1	1644.4–1650.1	ν_C=C_, stretching vibration of the cis R_1_HC=CHR_2_ groups
1455.7–1463.0	1455.9–1460.7	δ_CH_, bending vibration in the CH_2_ and CH_3_ groups
1412.4–1417.52	1415.9–1418.0	δ^rk^_=CH_, bending vibration (rocking) of the *cis* R_1_HC=CHR_2_ groups
1376.5–1379.4	1366.1–1370.6	δ_CH2_, bending vibration
1237.4–1241.6	1239.0–1243.3	δ_CH2_, bending vibration
1154.4–1162.9	1152.6–1154.4	δ_CH2_, bending vibration
1079.9–1098.7	1078.0–1080.2	ν_C-O_, stretching vibration
1052.7–1054.6	1051.4–1054.0	ν_C-O_, stretching vibration
1022.3–1034.6	1023.0–1025.1	ν_C-O_, stretching vibration
946.0–946.7	938.2–946.1	δ_C=C_, bending vibration of the *trans* R_3_HC=CHR_4_ groups

**Table 2:** Comparisons of the ATR-FTIR band assignments of the β-cyclodextrin hydrate and the corresponding β-cyclodextrin/lipid complexes.

Band Assignments for the β-CD (cm^−1^)	Band Assignments for the β-CD/Lipid Complexes (cm^−1^)	Description
3292.8–3294.3	3290.4–3309.2	ν_O-H_, stretching vibration
2923.6–2924.5	2921.1–2922.7	ν^as^_C-H_, asymmetric stretching vibration
1455.7–1463.0	1455.9–1460.7	δ_CH_, bending vibration in the CH_2_ and CH_3_ groups
1415.1–1416.3	1415.9–1418.0	δ_OH_, in-plan bending vibration
1365.7–1365.9	1366.1–1370.6	δ_CH2_, bending vibration
1334.1–1334.6	1331.3–1340.7	δ_CH_, bending vibration
1152.2–1152.3	1152.6–1154.4	δ_C-O-C_, stretching vibration for the glucosidic bonds
1076.9–1077.0	1078.0–1080.2	ν_C-O_, stretching vibration/ν_C-C_, stretching vibration
1021.4–1021.8	1023.0–1025.1	ν_C-O_, stretching vibration
937.8–937.8	938.2–946.1	ν_ring_, stretching vibration of the cyclodextrin ring
857.2–858.6	854.8–863.0	ν_α(1→4)_, stretching vibration of the α(1→4) glucosidic bond

## Data Availability

The data presented in this study are available in the Supplementary Materials file.

## Supplementary Materials

The following supporting information can be downloaded at: https://www.mdpi.com/article/10.3390/foods11223632/s1, Figure S1: TG–DTG analysis of the raw (unprocessed) chicken breast lipid fractions (duplicate samples “a” and “b”, codes “BUa”—green and “BUb”—red); Figure S2: TG–DTG analysis of the raw (unprocessed) chicken breast and thigh lipid fractions (duplicate samples “a”, codes “BUa”—green and “TUa”—red); Figure S3: TG–DTG analysis of the processed (boiling) chicken breast and thigh lipid fractions (single samples “a”, codes “BPa”—green and “TPa”—red); Figure S4: DSC analysis of the raw (unprocessed) chicken breast lipid fractions as duplicate samples “a” and “b” (codes “BUa”—green and “BUb”—red); Figure S5: DSC analysis of the raw (unprocessed) chicken breast and thigh lipid fractions (duplicate samples “a”, codes “BUa”—green and “TUa”—red); Figure S6: DSC analysis of the processed (boiling) chicken breast and thigh lipid fractions (single samples “a”, codes “BPa”—green and “TPa”—red); Figure S7: ATR-FTIR analysis of the raw (unprocessed) chicken breast and thigh lipid fractions (duplicate samples “a”, codes “BUa”—green and “TUa”—red); Figure S8: ATR-FTIR analysis of the processed (boiling) chicken breast and thigh lipid fractions (single samples “a”, codes “BPa”—green and “TPa”—red); Figure S9: Superimposed TG–DTG thermograms for the β-cyclodextrin hydrate as duplicate samples “a” and “b” (codes “Ka”—green and “Kb”—red); Figure S10: Superimposed TG–DTG thermograms for the β-cyclodextrin/raw breast chicken lipid complexes as duplicate samples “a” and “b” (codes “XBUa”—green and “XBUb”—red); Figure S11: Superimposed TG–DTG thermograms for the β-cyclodextrin/raw breast chicken lipid complex (blue), β-cyclodextrin hydrate (green), and raw breast chicken lipid fraction (red) (codes “XBU”, “K” and “BU”, duplicate samples “a”); Figure S12: Superimposed TG–DTG thermograms for the β-cyclodextrin/raw breast chicken lipid complex (blue), β-cyclodextrin hydrate (green), and raw breast chicken lipid fraction (red) (codes “XBU”, “K” and “BU”, duplicate samples “b”); Figure S13: Superimposed TG–DTG thermograms for the β-cyclodextrin/processed (boiling) breast chicken lipid complex (blue), β-cyclodextrin hydrate (green), and processed (boiling) breast chicken lipid fraction (red) (codes “XBP”, “K” and “BP”, single samples “a”); Figure S14: Superimposed TG–DTG thermograms for the β-cyclodextrin/raw thigh chicken lipid complex (blue), β-cyclodextrin hydrate (green), and raw thigh chicken lipid fraction (red) (codes “XTU”, “K” and “TU”, duplicate samples “a”); Figure S15: Superimposed TG–DTG thermograms for the β-cyclodextrin/processed (boiling) thigh chicken lipid complex (blue), β-cyclodextrin hydrate (green), and processed (boiling) thigh chicken lipid fraction (red) (codes “XTP”, “K” and “TP”, single samples “a”); Figure S16: Superimposed DSC curves for the β-cyclodextrinhydrate as duplicate samples “a” and “b” (codes “Ka”—green and “Kb”—red); Figure S17: Superimposed DSC curves for the β-cyclodextrin/raw breast chicken lipid complexes as duplicate samples “a” and “b” (codes “XBUa”—green and “XBUb”—red); Figure S18: Superimposed DSC curves for the β-cyclodextrin/raw breast chicken lipid complex (blue), β-cyclodextrin hydrate (green), and raw breast chicken lipid fraction (red) (codes “XBU”, “K” and “BU”, duplicate samples “a”); Figure S19: Superimposed DSC curves for the β-cyclodextrin/raw breast chicken lipid complex (blue), β-cyclodextrin hydrate (green), and raw breast chicken lipid fraction (red) (codes “XBU”, “K” and “BU”, duplicate samples “b”); Figure S20: Superimposed DSC curves for the β-cyclodextrin/processed (boiling) breast chicken lipid complex (blue), β-cyclodextrin hydrate (green), and processed (boiling) breast chicken lipid fraction (red) (codes “XBP”, “K” and “BP”, single samples “a”); Figure S21: Superimposed DSC curves for the β-cyclodextrin/raw thigh chicken lipid complex (blue), β-cyclodextrin hydrate (green), and raw thigh chicken lipid fraction (red) (codes “XTU”, “K” and “TU”, duplicate samples “a”); Figure S22: Superimposed ATR-FTIR spectra for β-cyclodextrin (top—magenta), lipid fraction from the unprocessed thigh chicken (middle—red) and β-cyclodextrin/unprocessed thigh chicken lipid complex (bottom—blue) (codes “K”, “TU” and “XTU”, duplicate samples “a”); Figure S23: Superimposed ATR-FTIR spectra for β-cyclodextrin (top—magenta), lipid fraction from the processed (boiling) thigh chicken (middle—red) and β-cyclodextrin/processed thigh chicken lipid complex (bottom—blue) (codes “K”, “TP” and “XTP”, single samples “a”); Table S1: PCA codes for the ATR-FTIR bands of the β-cyclodextrin/chicken lipid complexes, β-cyclodextrin hydrate and the non-complexed lipid fractions; band wavenumber variable codes contain “v” or “d” for stretching and bending vibrations of the corresponding bonds (in parenthesis), followed by the source (“K” for β-cyclodextrin and “L” for lipids); band intensity variable codes begin with “I”; Figure S24: PC_2_ versus PC_1_ loadings plot from the PCA analysis of all FTIR data (both intensities and wavenumbers) of the β-cyclodextrin/unprocessed and processed chicken breast and thigh lipid complexes, β-cyclodextrin and non-complexed lipid fractions; variable codes contain “v” or “d” for stretching and bending vibrations of the corresponding bonds (in parenthesis), followed by the source (“K” for β-cyclodextrin and “L” for lipids); band intensity variable codes begin with “I” (e.g., “v4(CO)_KL” and “I_v4(CO)_KL” stand for the variable corresponding to the wavenumber and intensity of the 4th stretching vibration of the C-O groups at ~1025 cm^−1^ in both β-cyclodextrin and lipids, respectively); Figure S25: PC_3_ versus PC_1_ loadings plot from the PCA analysis of all FTIR data (both intensities and wavenumbers) of the β-cyclodextrin/unprocessed and processed chicken breast and thigh lipid complexes, β-cyclodextrin and non-complexed lipid fractions; variable codes contain “v” or “d” for stretching and bending vibrations of the corresponding bonds (in parenthesis), followed by the source (“K” for β-cyclodextrin and “L” for lipids); band intensity variable codes begin with “I” (e.g., “v2(CO)_L” and “I_v2(CO)_L” stand for the variable corresponding to the wavenumber and intensity of the 2nd stretching vibration of the CO groups at ~1089 cm^−1^ in lipids, respectively); Figure S26: PC_2_ versus PC_1_ loadings plot from the PCA analysis of FTIR band intensity data of the β-cyclodextrin/unprocessed and processed chicken breast and thigh lipid complexes, β-cyclodextrin and non-complexed lipid fractions; variable codes contain “v” or “d” for stretching and bending vibrations of the corresponding bonds (in parenthesis), followed by the source (“K” for β-cyclodextrin and “L” for lipids); band intensity variable codes begin with “I” (e.g., “I_v4(CO)_KL” stands for the variable corresponding to the intensity of the 4th stretching vibration of the C-O groups at ~1025 cm^−1^ in both β-cyclodextrin and lipids); Figure S27: PC_3_ versus PC_1_, PC_2_ scores plot from the PCA analysis of the FTIR band wavenumber data of the β-cyclodextrin/unprocessed and processed chicken breast and thigh lipid complexes (codes “XBU”, “XBP”, “XTU” and “XTP”), in comparison with β-cyclodextrin (code “K”) and non-complexed lipid fractions (codes “BU”, “BP”, “TU” and “TP”); “a” and “b” stand for duplicate samples; Figure S28: PC_2_ versus PC_1_ loadings plot from the PCA analysis of the FTIR band wavenumber data of the β-cyclodextrin/unprocessed and processed chicken breast and thigh lipid complexes, β-cyclodextrin and non-complexed lipid fractions; variable codes contain “v” or “d” for stretching and bending vibrations of the corresponding bonds (in parenthesis), followed by the source (“K” for β-cyclodextrin and “L” for lipids); e.g., “v4(CO)_KL” stands for the variable corresponding to the wavenumber of the 4th stretching vibration of the C-O groups at ~1025 cm^−1^ in both β-cyclodextrin and lipids; Figure S29: PC_3_ versus PC_1_, PC_2_ scores plot from the PCA analysis of the selected FTIR band wavenumber data (see the text from the article) of the β-cyclodextrin/unprocessed and processed chicken breast and thigh lipid complexes (codes “XBU”, “XBP”, “XTU” and “XTP”), in comparison with β-cyclodextrin (code “K”) and non-complexed lipid fractions (codes “BU”, “BP”, “TU” and “TP”); “a” and “b” stand for duplicate samples; Figure S30: Residual variance from the PCA analysis of the selected FTIR band wavenumber data (see the text from the article) of the β-cyclodextrin/unprocessed and processed chicken breast and thigh lipid complexes, β-cyclodextrin and non-complexed lipid fractions.
