# Supplementary material for: FTIR–PCA Approach on Raw and Thermally Processed Chicken Lipids Stabilized by Nano-Encapsulation in β-Cyclodextrin
Source: Foods. 2022 Nov 14;11(22):3632. doi: 10.3390/foods11223632 (PMC9689604; doi:10.3390/foods11223632)
Supplement: Supplementary file 1 [file foods-11-03632-s001.zip › foods-1988882-supplementary.pdf]

Type of the Paper (Article)

Supplementary material

FTIR–PCA Approach on Raw and Thermally Processed Chicken Lipids Stabilized by Nano-encapsulation in β-Cyclodextrin

1. Thermogravimetry – differential thermogravimetry (TG-DTG) for the chicken lipid samples

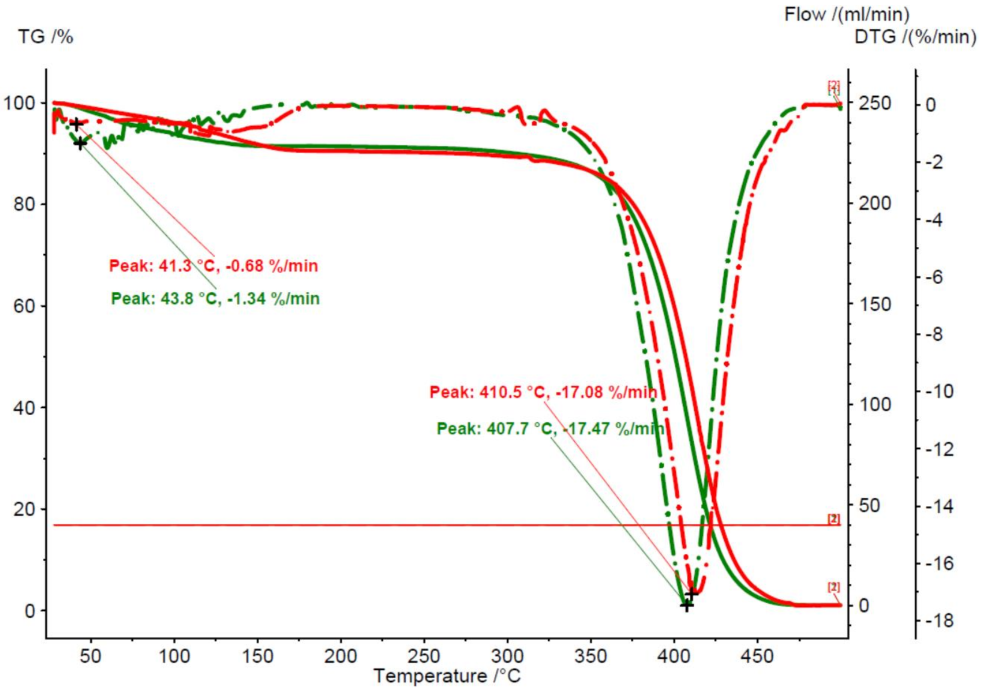

Figure S1. TG-DTG analysis of the raw (unprocessed) chicken breast lipid fractions (duplicate samples “a” and “b”, codes “BUa” – green and “BUb” – red)

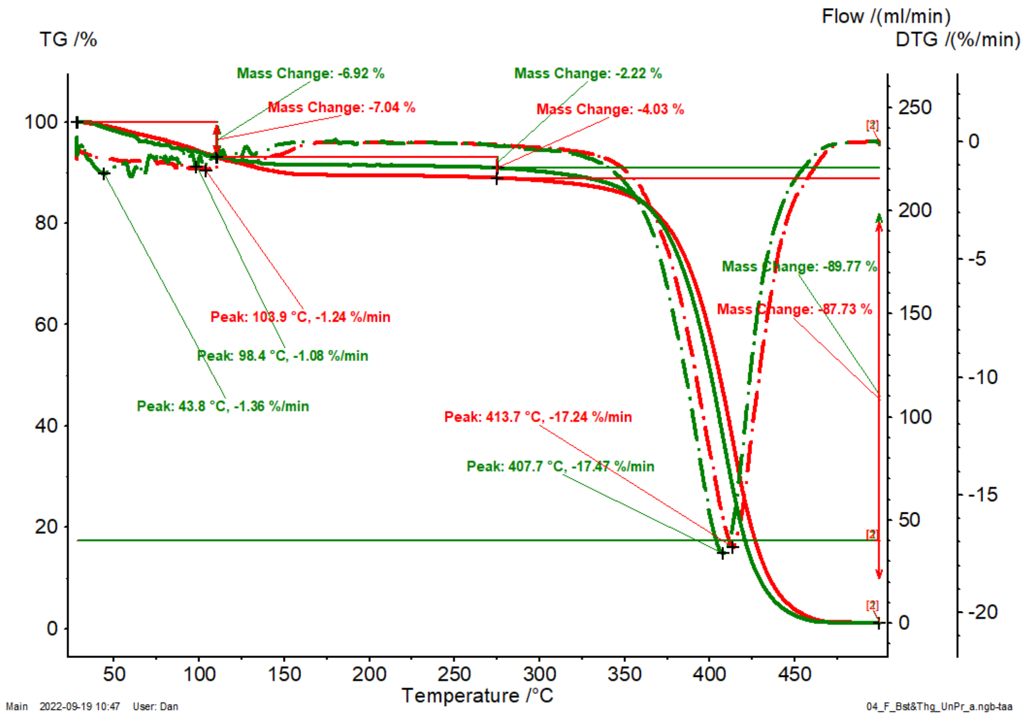

Figure S2. TG-DTG analysis of the raw (unprocessed) chicken breast and thigh lipid fractions (duplicate samples "a", codes "BUa" – green and "TUa" – red)

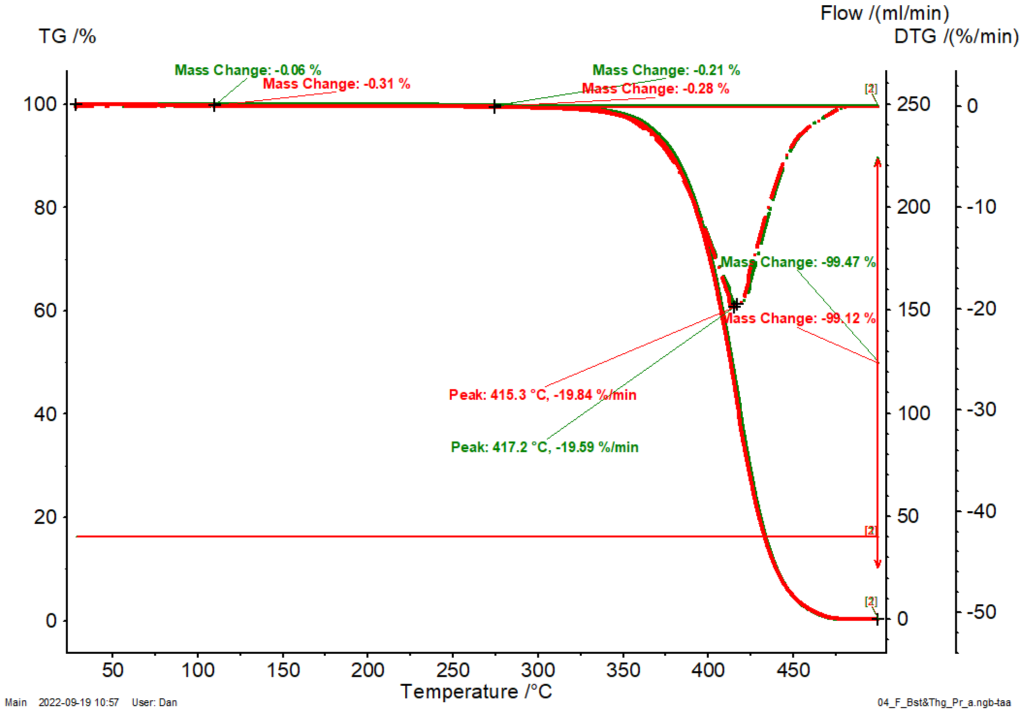

Figure S3. TG-DTG analysis of the processed (boiling) chicken breast and thigh lipid fractions (single samples "a", codes "BPa" – green and "TPa" – red)

2. Differential scanning calorimetry (DSC) for the chicken lipid samples

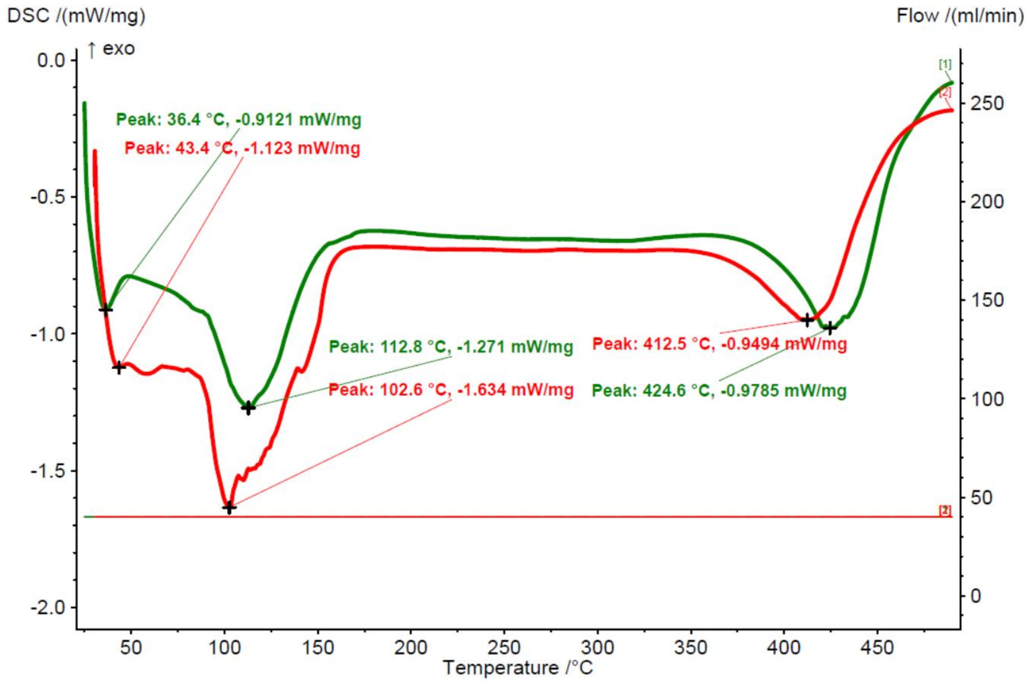

Figure S4. DSC analysis of the raw (unprocessed) chicken breast lipid fractions as duplicate samples "a" and "b" (codes "BUa" – green and "BUb" – red)

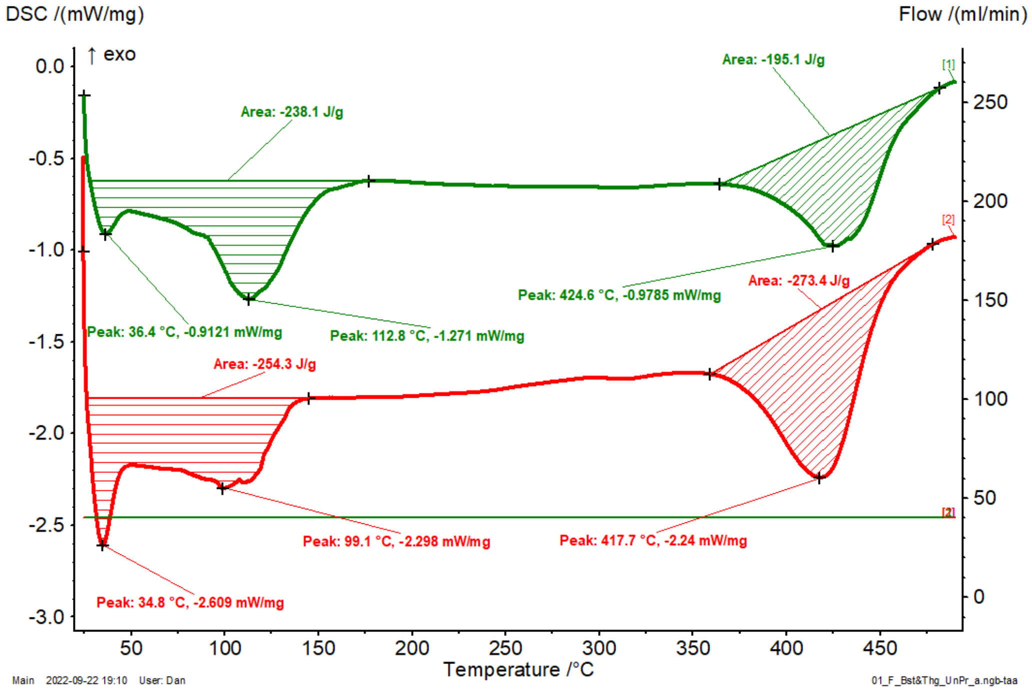

Figure S5. DSC analysis of the raw (unprocessed) chicken breast and thigh lipid fractions (duplicate samples "a", codes "BUa" – green and "TUa" – red)

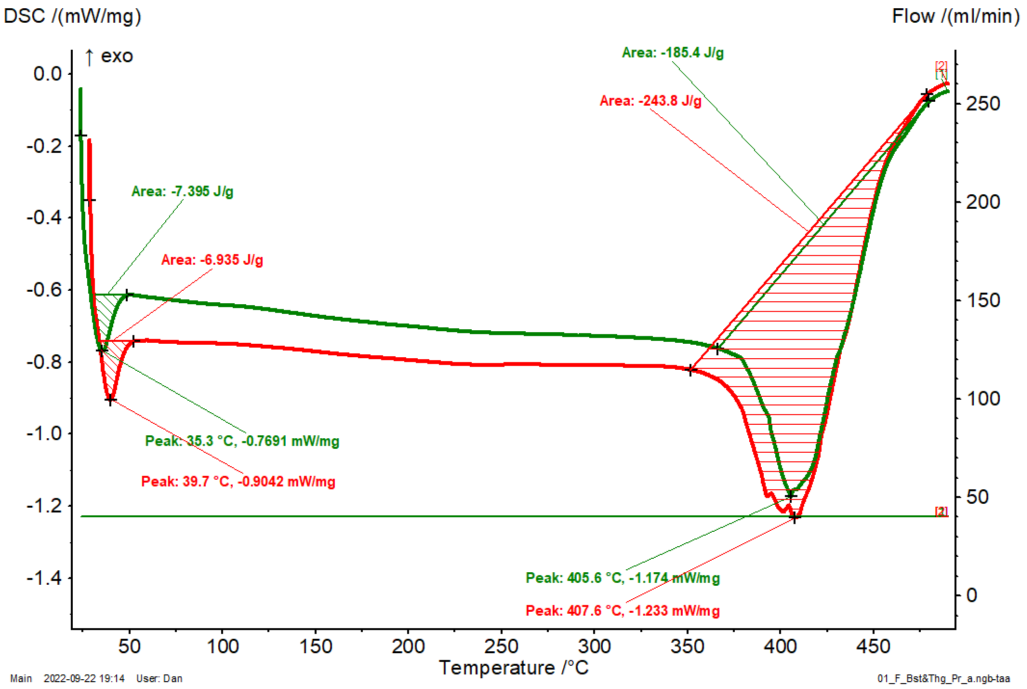

Figure S6. DSC analysis of the processed (boiling) chicken breast and thigh lipid fractions (single samples “a”, codes “BPa” – green and “TPa” – red)

3. Attenuated total reflectance – Fourier transform infrared spectroscopy (ATR-FTIR) for the chicken lipid samples

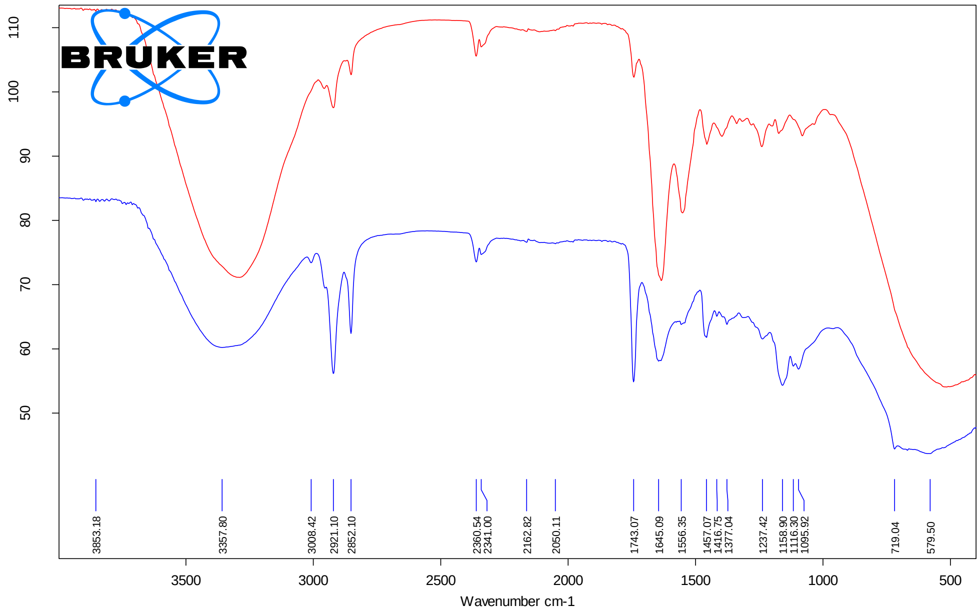

Figure S7. ATR-FTIR analysis of the raw (unprocessed) chicken breast and thigh lipid fractions (duplicate samples "a", codes "BUa" – green and "TUa" – red)

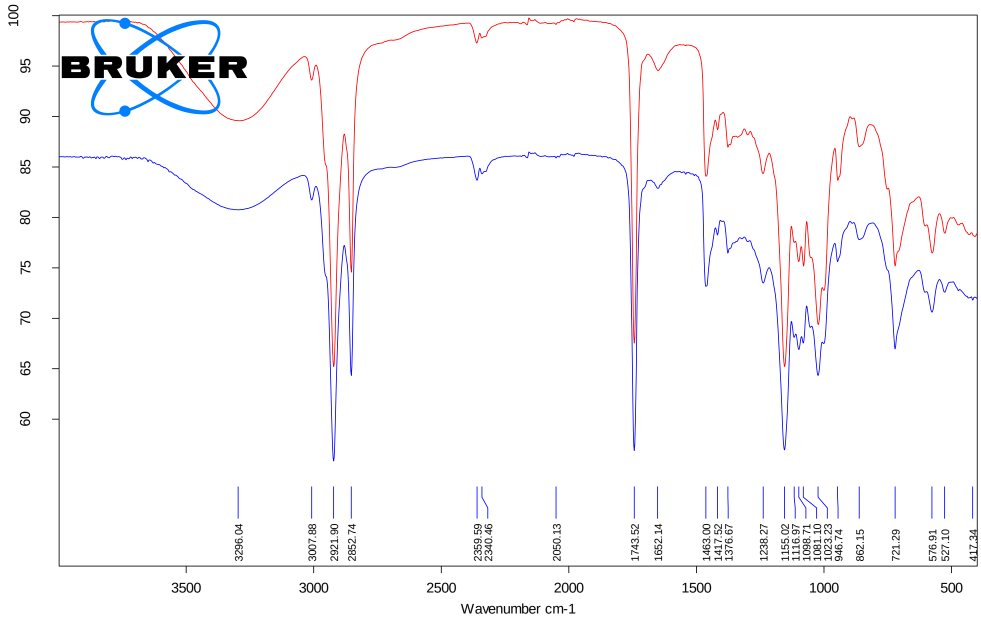

Figure S8. ATR-FTIR analysis of the processed (boiling) chicken breast and thigh lipid fractions (single samples "a", codes "BPa" – green and "TPa" – red)

4. Thermogravimetry – differential thermogravimetry (TG-DTG) for the β-cyclodextrin / chicken lipid complexes

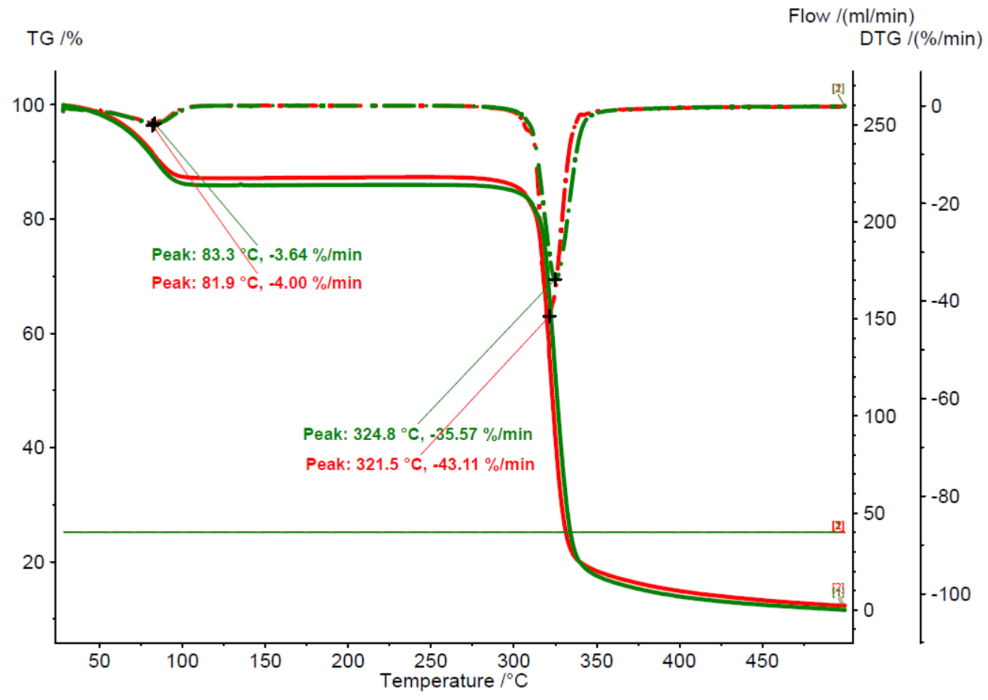

Figure S9. Superimposed TG-DTG thermograms for the β-cyclodextrin hydrate as duplicate samples "a" and "b" (codes "Ka" - green and "Kb" – red)

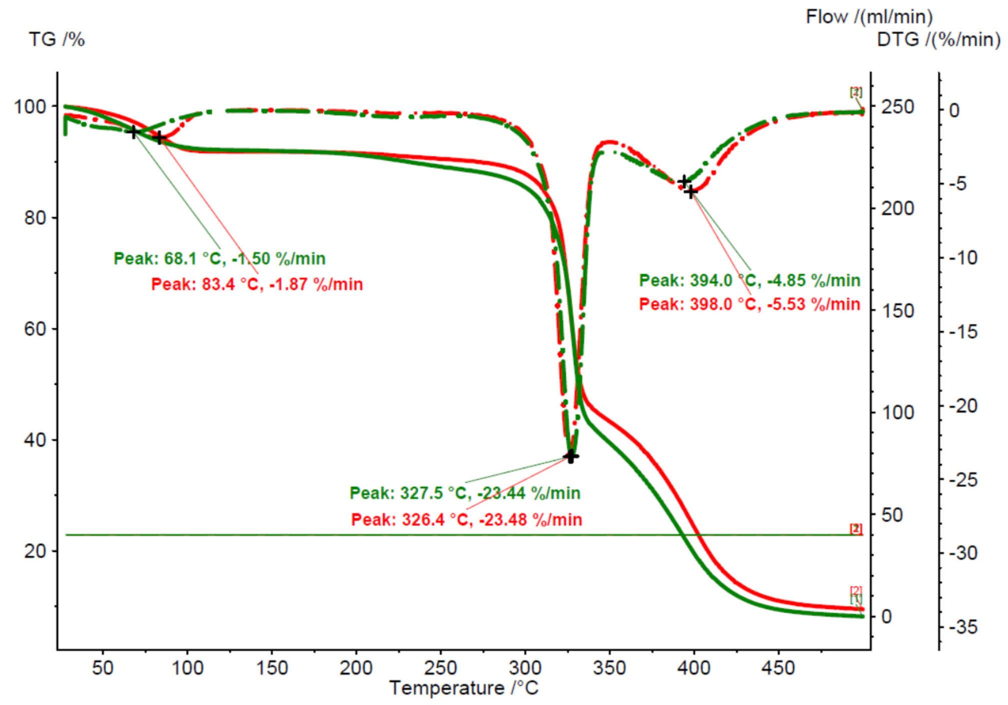

Figure S10. Superimposed TG-DTG thermograms for the β-cyclodextrin/raw breast chicken lipid complexes as duplicate samples "a" and "b" (codes "XBUa" – green and "XBUb" – red)

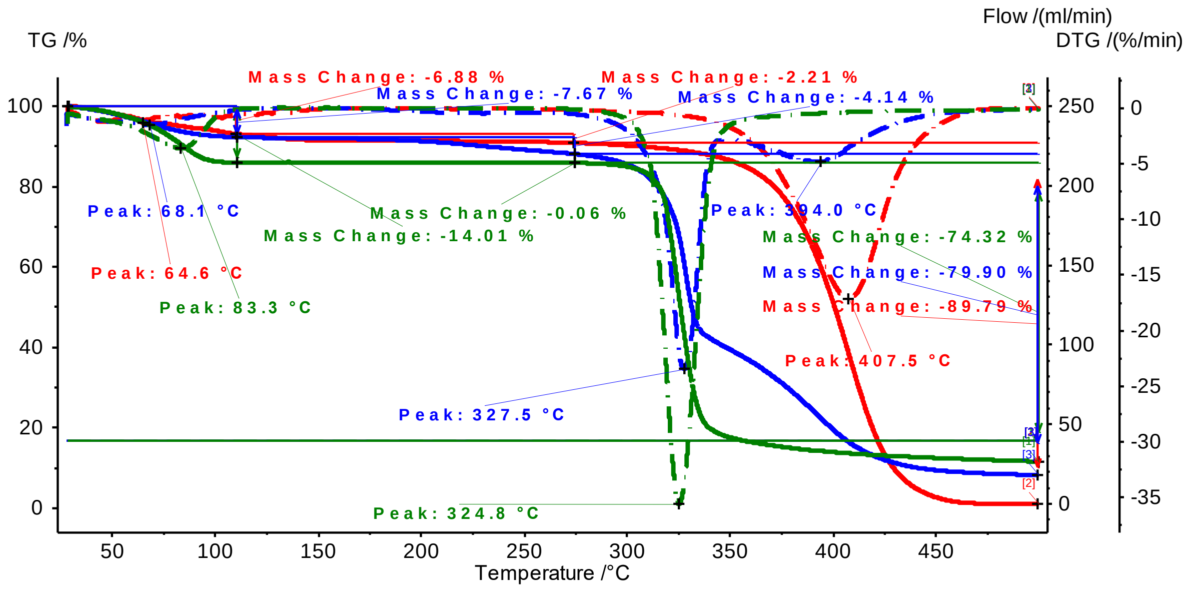

Figure S11. Superimposed TG-DTG thermograms for the β-cyclodextrin/raw breast chicken lipid complex (blue), β-cyclodextrin hydrate (green), and raw breast chicken lipid fraction (red) (codes "XBU", "K" and "BU", duplicate samples "a")

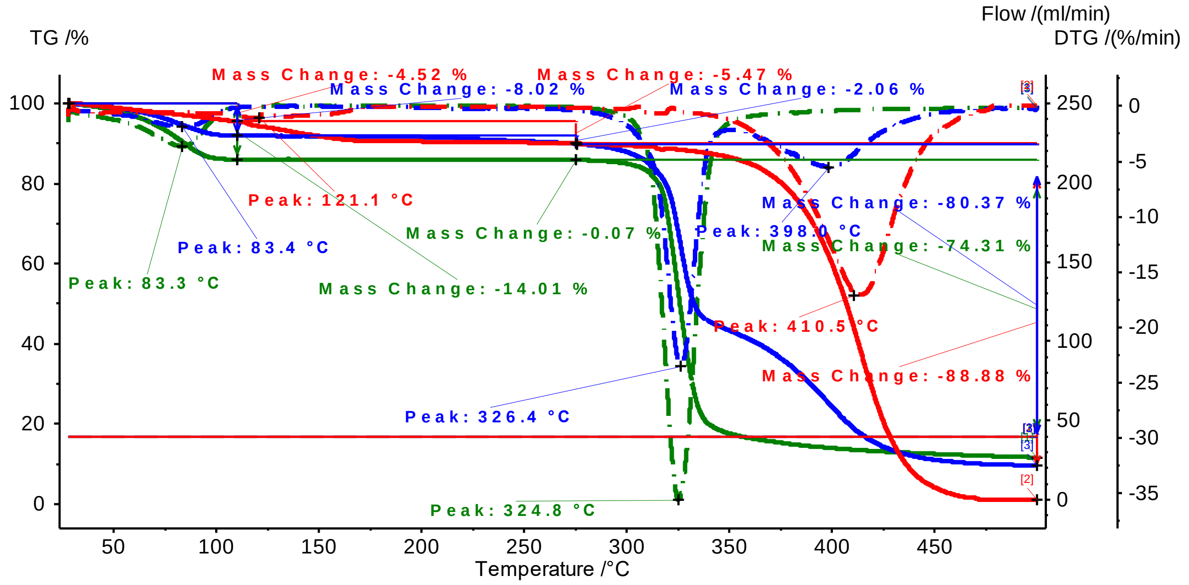

Figure S12. Superimposed TG-DTG thermograms for the β-cyclodextrin/raw breast chicken lipid complex (blue), β-cyclodextrin hydrate (green), and raw breast chicken lipid fraction (red) (codes "XBU", "K" and "BU", duplicate samples "b")

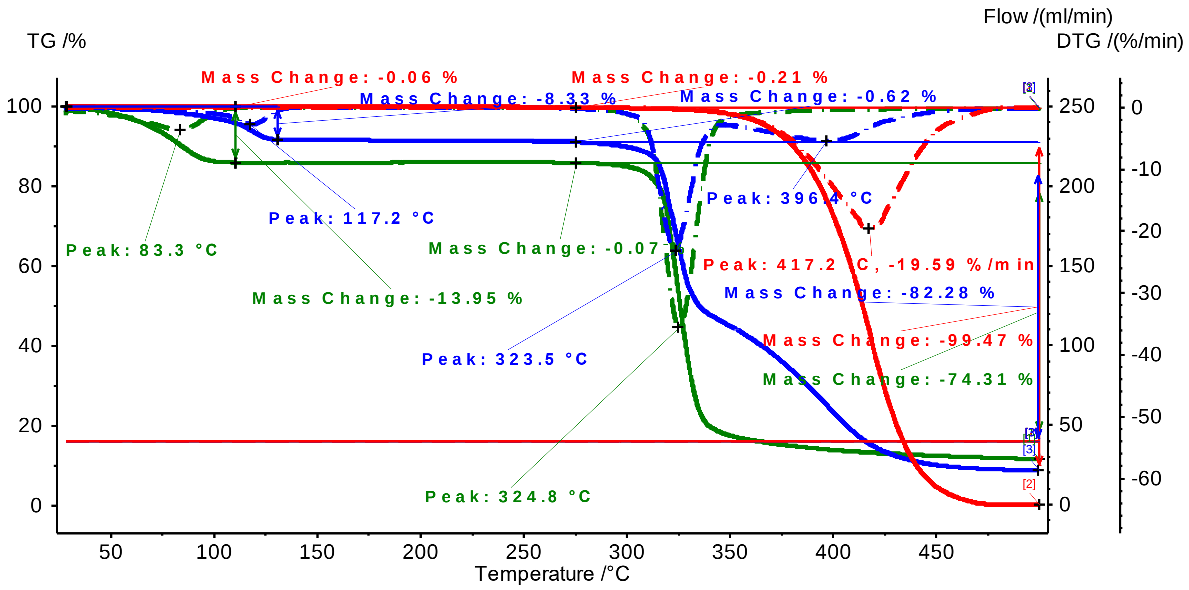

Figure S13. Superimposed TG-DTG thermograms for the β-cyclodextrin/processed (boiling) breast chicken lipid complex (blue), β-cyclodextrin hydrate (green), and processed (boiling) breast chicken lipid fraction (red) (codes "XBP", "K" and "BP", single samples "a")

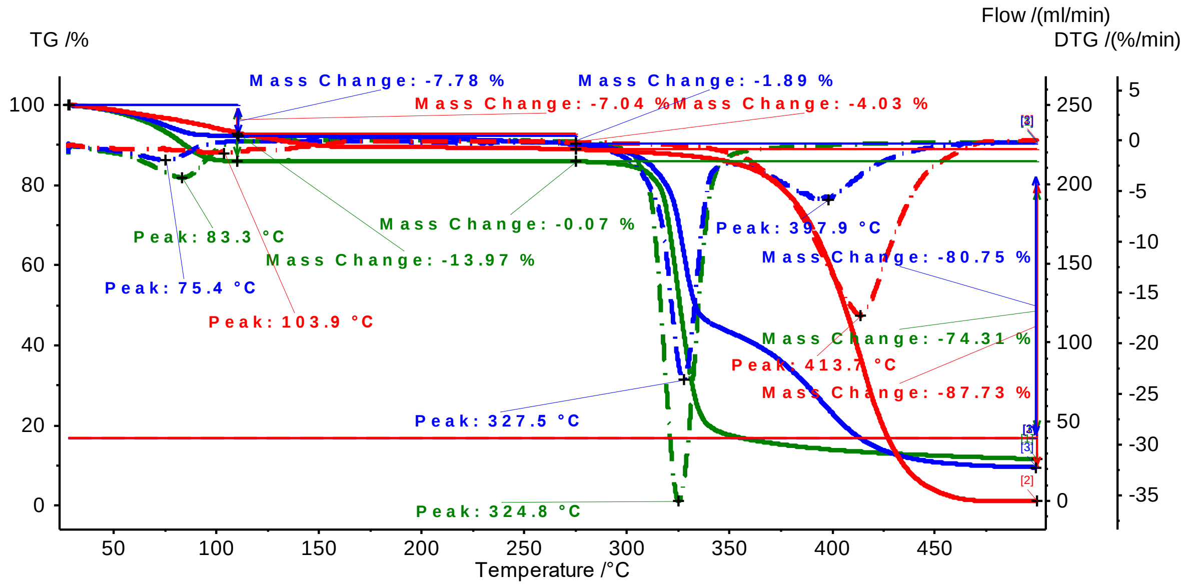

Figure S14. Superimposed TG-DTG thermograms for the β-cyclodextrin/raw thigh chicken lipid complex (blue), β-cyclodextrin hydrate (green), and raw thigh chicken lipid fraction (red) (codes "XTU", "K" and "TU", duplicate samples "a")

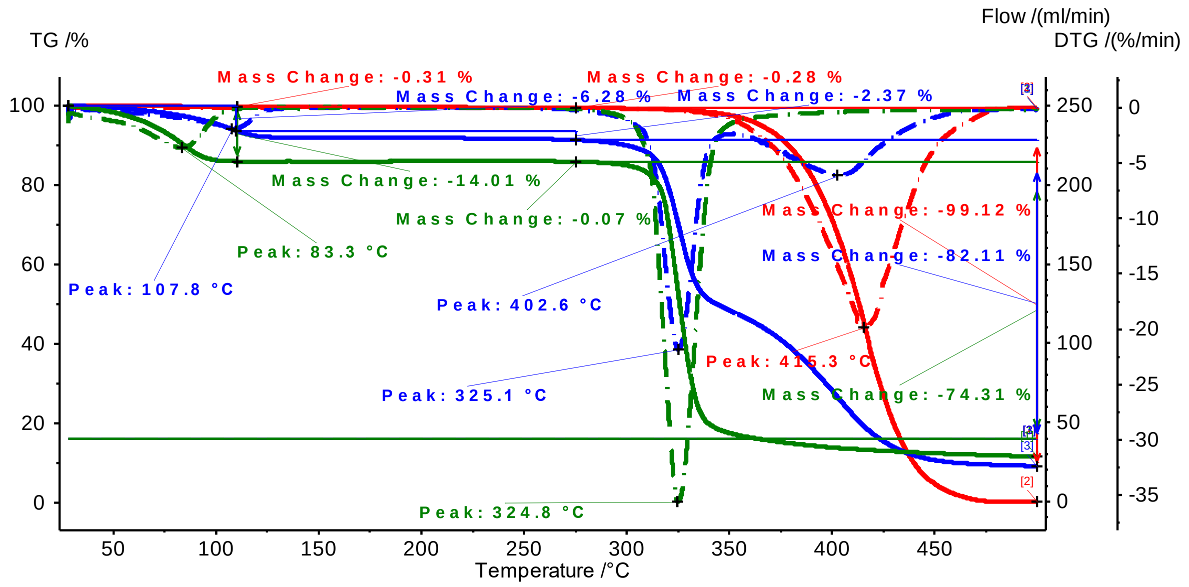

Figure S15. Superimposed TG-DTG thermograms for the β-cyclodextrin/processed (boiling) thigh chicken lipid complex (blue), β-cyclodextrin hydrate (green), and processed (boiling) thigh chicken lipid fraction (red) (codes "XTP", "K" and "TP", single samples "a")

5. Differential scanning calorimetry (DSC) for the β-cyclodextrin / chicken lipid complexes

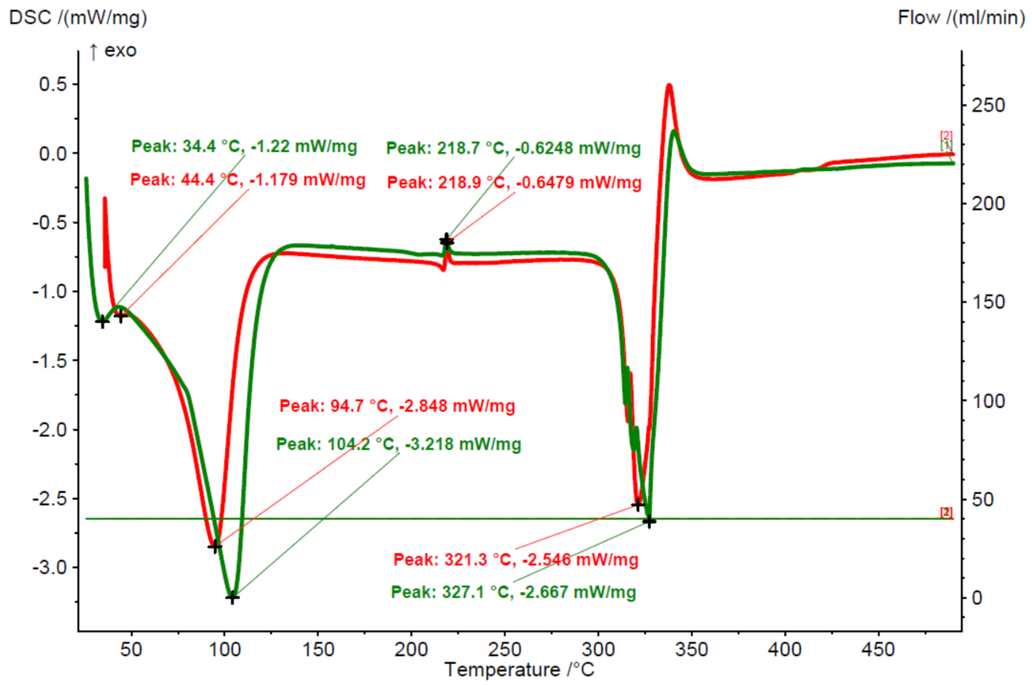

Figure S16. Superimposed DSC curves for the β-cyclodextrinhydrate as duplicate samples "a" and "b" (codes "Ka" – green and "Kb" – red)

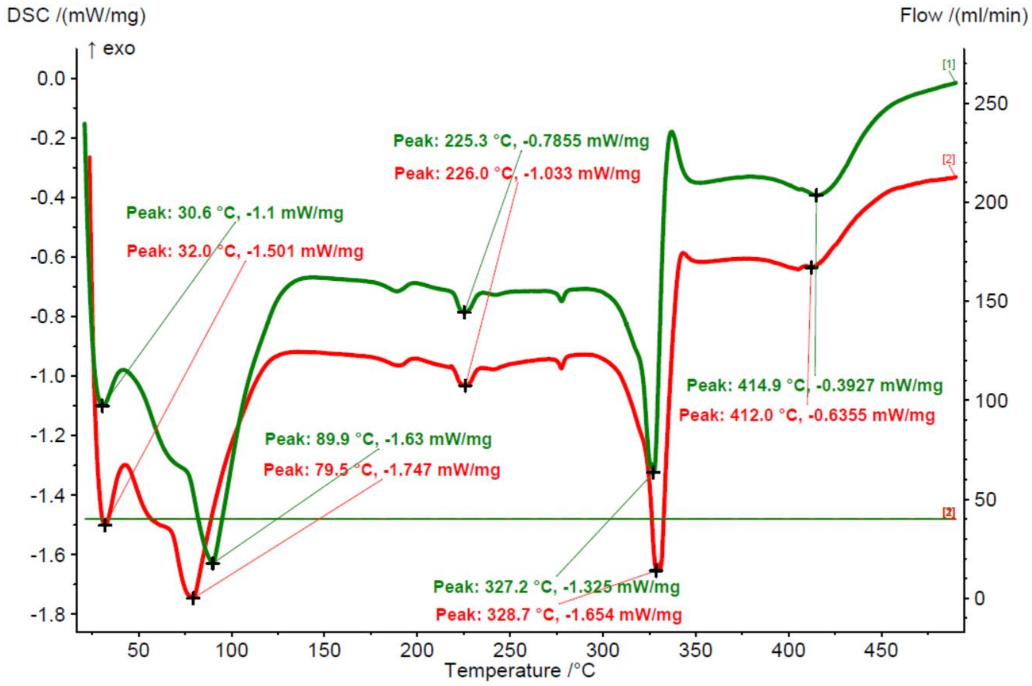

Figure S17. Superimposed DSC curves for the β-cyclodextrin/raw breast chicken lipid complexes as duplicate samples "a" and "b" (codes "XBUa" – green and "XBUb" – red)

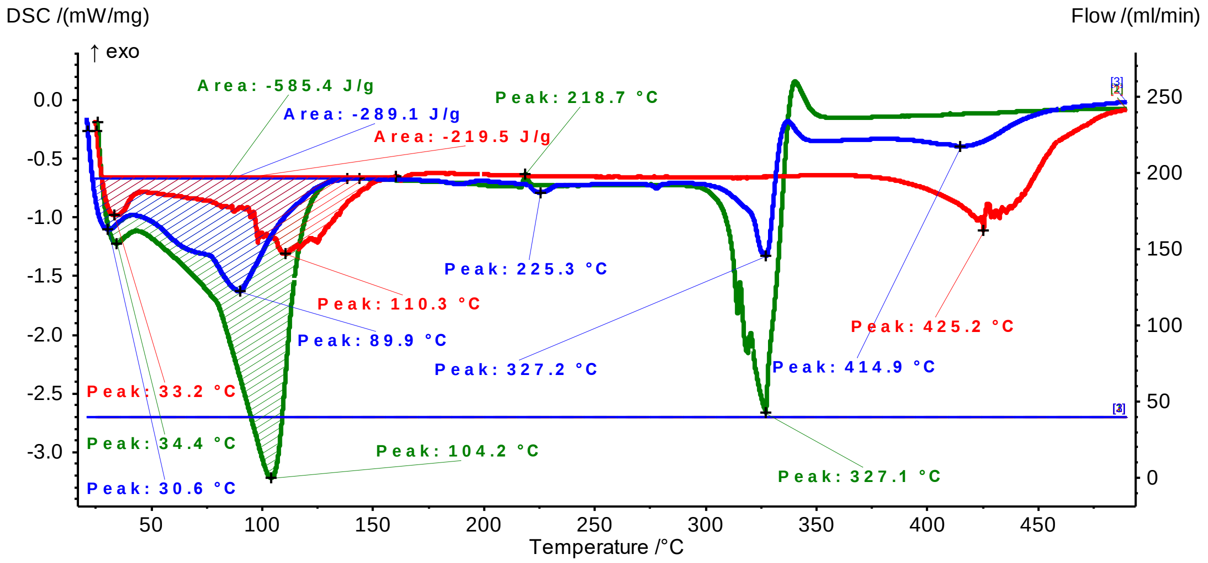

Figure S18. Superimposed DSC curves for the β-cyclodextrin/raw breast chicken lipid complex (blue), β-cyclodextrin hydrate (green), and raw breast chicken lipid fraction (red) (codes "XBU", "K" and "BU", duplicate samples "a")

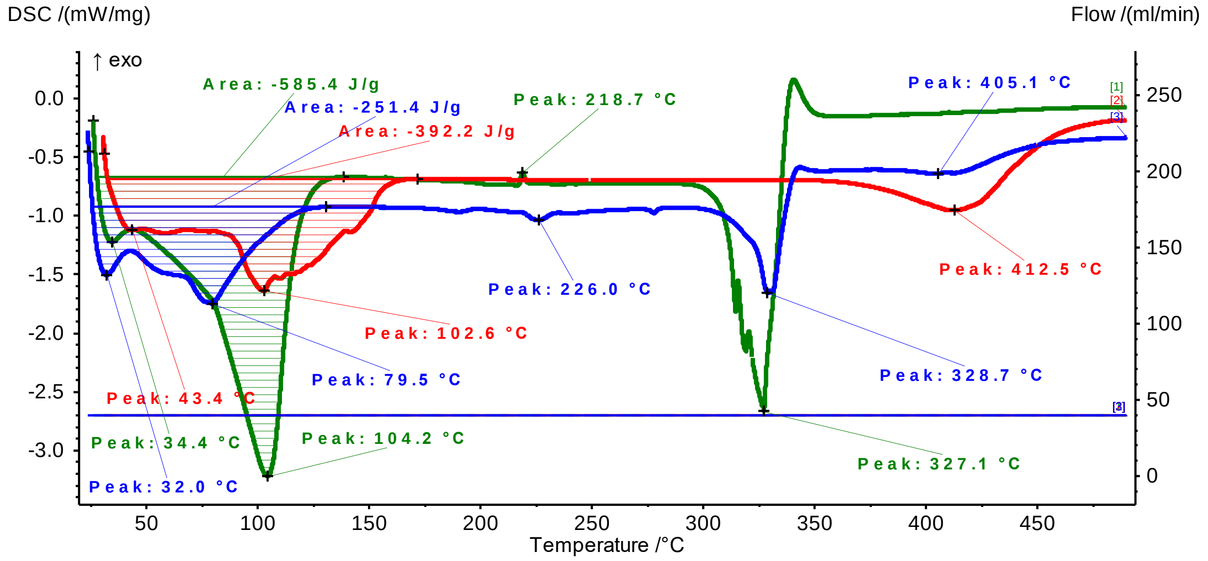

Figure S19. Superimposed DSC curves for the β-cyclodextrin/raw breast chicken lipid complex (blue), β-cyclodextrin hydrate (green), and raw breast chicken lipid fraction (red) (codes "XBU", "K" and "BU", duplicate samples "b")

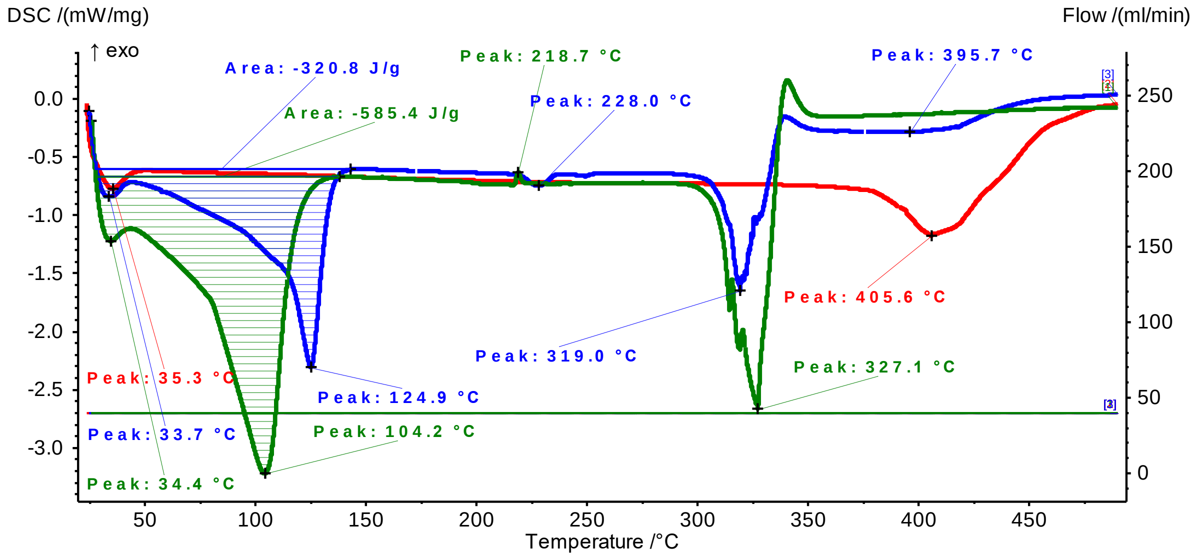

Figure S20. Superimposed DSC curves for the β-cyclodextrin/processed (boiling) breast chicken lipid complex (blue), β-cyclodextrin hydrate (green), and processed (boiling) breast chicken lipid fraction (red) (codes "XBP", "K" and "BP", single samples "a")

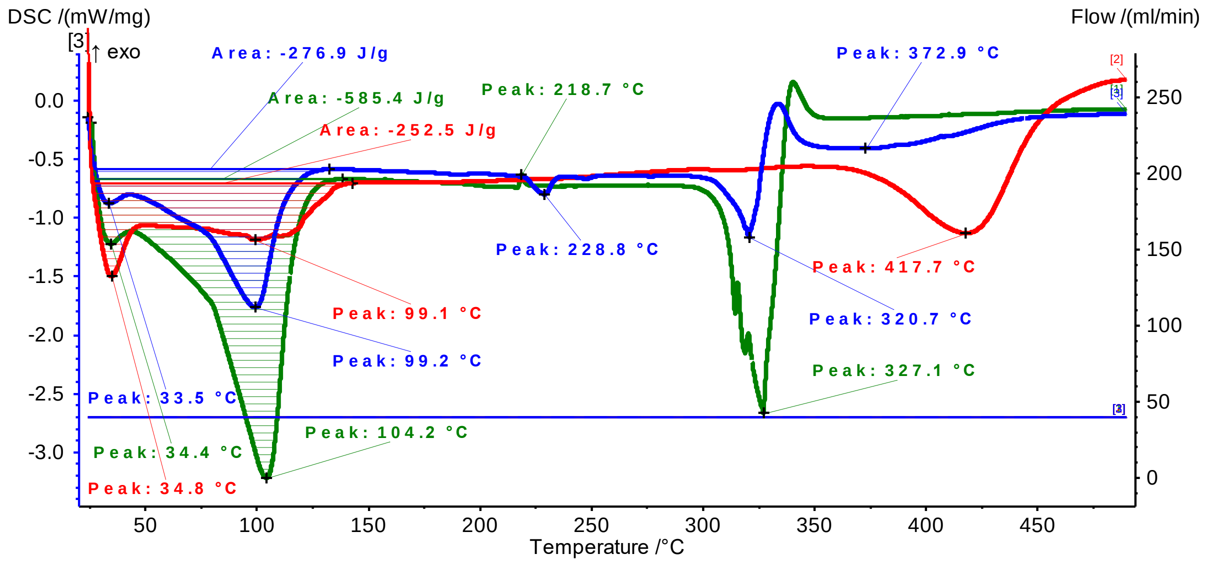

Figure S21. Superimposed DSC curves for the β-cyclodextrin/raw thigh chicken lipid complex (blue), β-cyclodextrin hydrate (green), and raw thigh chicken lipid fraction (red) (codes "XTU", "K" and "TU", duplicate samples "a")

6. Attenuated total reflectance – Fourier transform infrared spectroscopy (ATR-FTIR) for the β-cyclodextrin / chicken lipid complexes

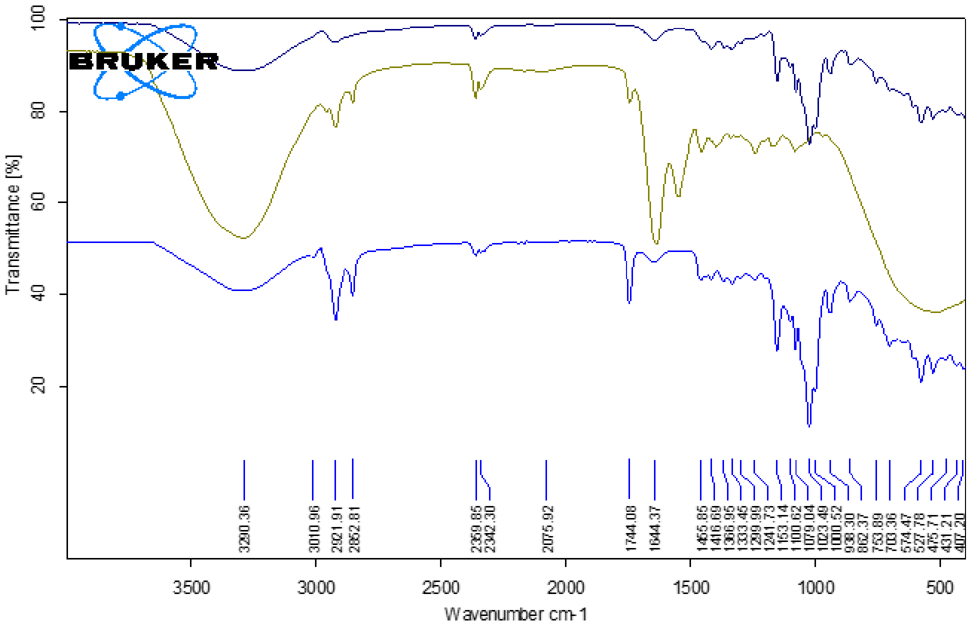

Figure S22. Superimposed ATR-FTIR spectra for β-cyclodextrin (top - magenta), lipid fraction from the unprocessed thigh chicken (middle - red) and β-cyclodextrin / unprocessed thigh chicken lipid complex (bottom - blue) (codes "K", "TU" and "XTU", duplicate samples "a")

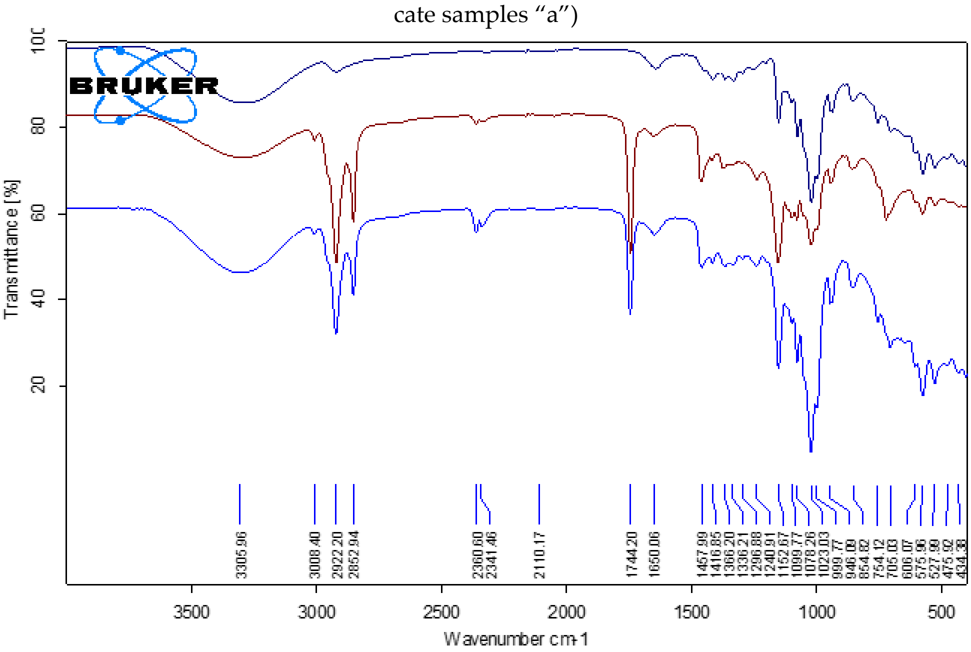

Figure S23. Superimposed ATR-FTIR spectra for β-cyclodextrin (top - magenta), lipid fraction from the processed (boiling) thigh chicken (middle - red) and β-cyclodextrin / processed thigh chicken lipid complex (bottom - blue) (codes "K", "TP" and "XTP", single samples "a")

7. Principal component analysis (PCA) of the ATR-FTIR data for the β-cyclodextrin / chicken lipid complexes

Table S1. PCA codes for the ATR-FTIR bands of the β-cyclodextrin/chicken lipid complexes, β-cyclodextrin hydrate and the non-complexed lipid fractions; band wavenumber variable codes contain "v" or "d" for stretching and bending vibrations of the corresponding bonds (in parenthesis), followed by the source ("K" for β-cyclodextrin and "L" for lipids); band intensity variable codes start with "I"

Code (wavenumber / intensity)	Approximate band assignments from β-CD ("K"), lipids ("L") or both components, ("KL") (cm^{-1})	Description
v(OH)_KL / I_v(OH)_KL	3299	$\nu_{O\text{-}H}$, stretching vibration from both β-CD and lipids
v(=CH)_L / I_v(=CH)_L	3009	$\nu^{s}_{=CH}$, symmetric stretching vibration from lipids
v1(CH)_L / I_v1(CH)_L	2954	$\nu^{as}_{C\text{-}H}$, asymmetric stretching vibration (1st band) from lipids
v2(CH)_KL / I_v2(CH)_KL	2922	$\nu^{as}_{C\text{-}H}$, asymmetric stretching vibration (2nd band) from both β-CD and lipids
v3(CH)_L / I_v3(CH)_L	2852	ν^{as}_{CH}, asymmetric stretching vibration (3rd band) from lipids
v(C=O)_L / I_v(C=O)_L	1743	$\nu_{C=O}$, stretching vibration of the ester groups from lipids
d(OH) or v(cis,C=C)_KL / I_d(OH) or I_v(cis,C=C)_KL	1645	δ_{CH}, bending vibration in the CH_2 and CH_3 groups / $\nu_{C=C}$, stretching vibration of the *cis* $R_1HC=CHR_2$ groups from β-CD and/or lipids
d(CH)_L / I_d(CH)_L	1458	δ_{CH}, bending vibration in the CH_2 and CH_3 groups from lipids
d(=CH) or d(OH)_KL / I_d(=CH) or I_d(OH)_KL	1416	$\delta^{rk}_{=CH}$, bending vibration (rocking) of the *cis* $R_1HC=CHR_2$ groups from lipids / δ_{OH}, in-plan bending vibration from β-CD
d1(CH/CH2)_KL / I_d1(CH/CH2)_KL	1372	δ_{CH2}, bending vibration (1st band) from both β-CD and lipids
d2(CH)_K / I_d2(CH)_K	1335	δ_{CH}, bending vibration (2nd band) from β-CD
d3(CH2)_L / I_d3(CH2)_L	1240	δ_{CH2}, bending vibration (3rd band) from lipids
d4(CH2) or d(COC-Glu)_KL / I_d4(CH2) or I_d(COC-Glu)_KL	1155	δ_{CH2}, bending vibration (4th band) from both β-CD and lipids / $\delta_{C\text{-}O\text{-}C}$, stretching vibration from β-CD
v1(CO)_L / I_v1(CO)_L	1117	$\nu_{C\text{-}O}$, stretching vibration (1st band) from lipids
v2(CO)_L / I_v2(CO)_L	1089	$\nu_{C\text{-}O}$, stretching vibration (2nd band) from lipids
v(CC)_K / I_v(CC)_K	1079	$\nu_{C\text{-}C}$, stretching vibration from β-CD
v3(CO)_L / I_v3(CO)_L	1053	$\nu_{C\text{-}O}$, stretching vibration (3rd band) from lipids
v4(CO)_KL / I_v4(CO)_KL	1025	$\nu_{C\text{-}O}$, stretching vibration (4th band) from both β-CD and lipids
d(trans,C=C)_L / I_d(trans,C=C)_L	946	$\delta_{C=C}$, bending vibration of the *trans* $R_3HC=CHR_4$ groups from lipids
v(Cy)_K / I_v(Cy)_K	941	ν_{ring}, stretching vibration of the cyclodextrin ring
v(aGly)_K / I_v(aGly)_K	859	$\nu_{\alpha(1\rightarrow4)}$, stretching vibration of the $\alpha(1\rightarrow4)$ glucosidic bond from β-CD
do(CH)_L / I_do(CH)_L	720	$\delta_{C\text{-}H}$, out-of-plane deformation from lipids

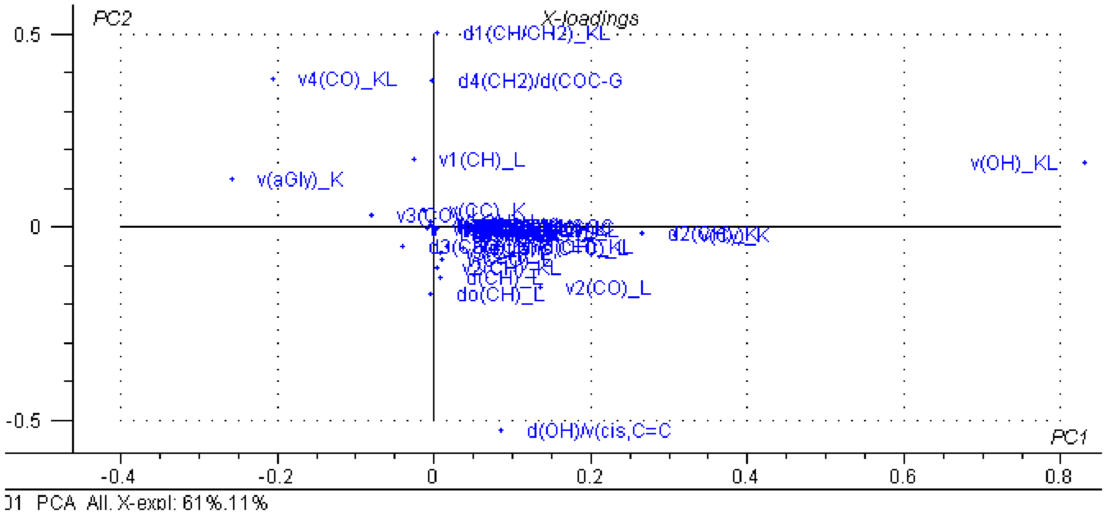

Figure S24. PC$_2$ versus PC$_1$ loadings plot from the PCA analysis of all FTIR data (both intensities and wavenumbers) of the β-cyclodextrin/unprocessed and processed chicken breast and thigh lipid complexes, β-cyclodextrin and non-complexed lipid fractions; variable codes contain "v" or "d" for stretching and bending vibrations of the corresponding bonds (in parenthesis), followed by the source ("K" for β-cyclodextrin and "L" for lipids); band intensity variable codes start with "I" (e.g., "v4(CO)_KL" and "I_v4(CO)_KL" stand for the variable corresponding to the wavenumber and intensity of the 4th stretching vibration of the C-O groups at ~1025 cm^{-1} in both β-cyclodextrin and lipids, respectively)

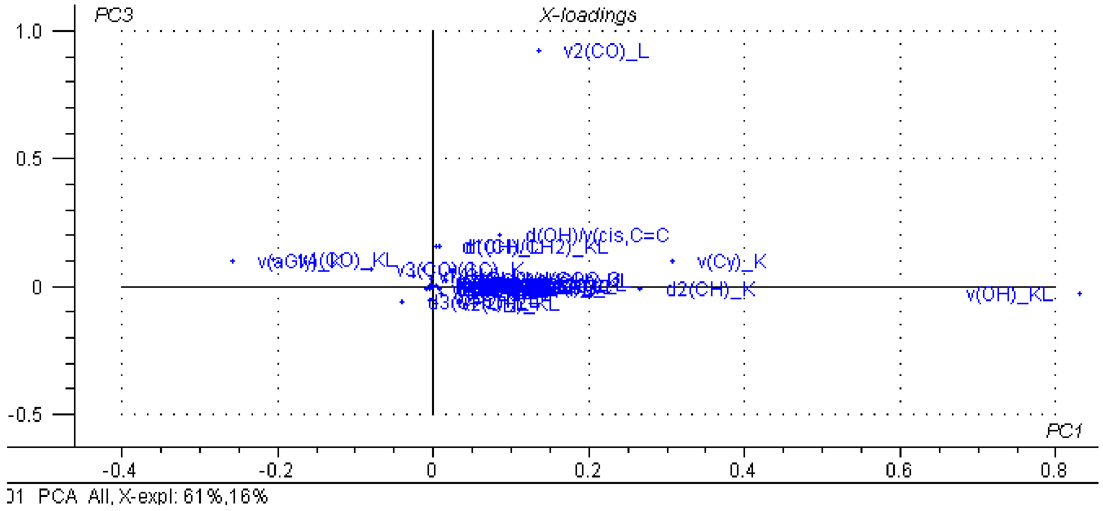

Figure S25. PC$_3$ versus PC$_1$ loadings plot from the PCA analysis of all FTIR data (both intensities and wavenumbers) of the β-cyclodextrin/unprocessed and processed chicken breast and thigh lipid complexes, β-cyclodextrin and non-complexed lipid fractions; variable codes contain "v" or "d" for stretching and bending vibrations of the corresponding bonds (in parenthesis), followed by the source ("K" for β-cyclodextrin and "L" for lipids); band intensity variable codes start with "I" (e.g., "v2(CO)_L" and "I_v2(CO)_L" stand for the variable corresponding to the wavenumber and intensity of the 2nd stretching vibration of the CO groups at ~1089 cm^{-1} in lipids, respectively)

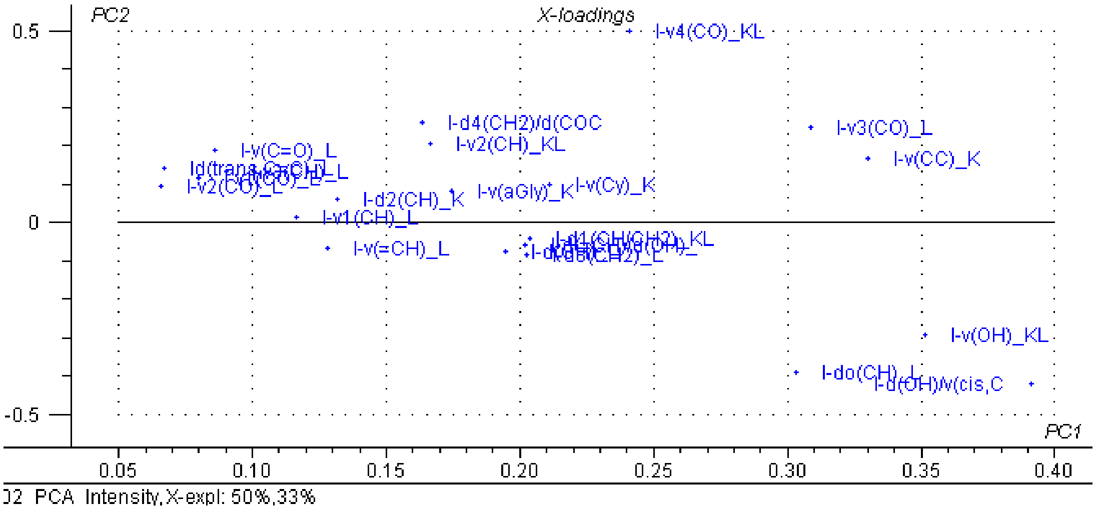

Figure S26. PC_2 versus PC_1 loadings plot from the PCA analysis of FTIR band intensity data of the β-cyclodextrin/unprocessed and processed chicken breast and thigh lipid complexes, β-cyclodextrin and non-complexed lipid fractions; variable codes contain "v" or "d" for stretching and bending vibrations of the corresponding bonds (in parenthesis), followed by the source ("K" for β-cyclodextrin and "L" for lipids); band intensity variable codes start with "I" (e.g., "I_v4(CO)_KL" stands for the variable corresponding to the intensity of the 4th stretching vibration of the C-O groups at ~1025 cm^{-1} in both β-cyclodextrin and lipids)

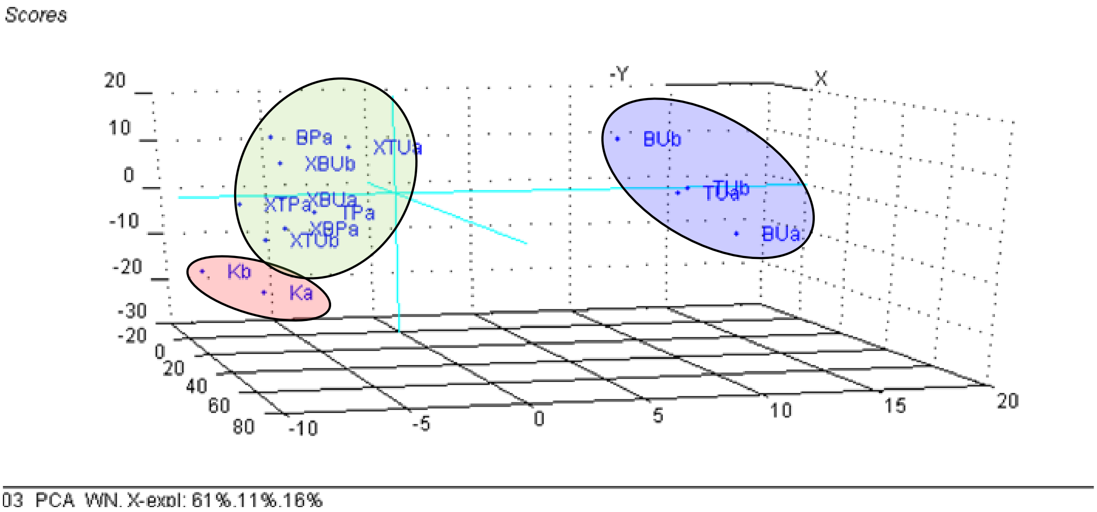

Figure S27. PC_3 versus PC_1, PC_2 scores plot from the PCA analysis of the FTIR band wavenumber data of the β-cyclodextrin/unprocessed and processed chicken breast and thigh lipid complexes (codes "XBU", "XBP", "XTU" and "XTP"), in comparison with β-cyclodextrin (code "K") and non-complexed lipid fractions (codes "BU", "BP", "TU" and "TP"); "a" and "b" stand for duplicate samples

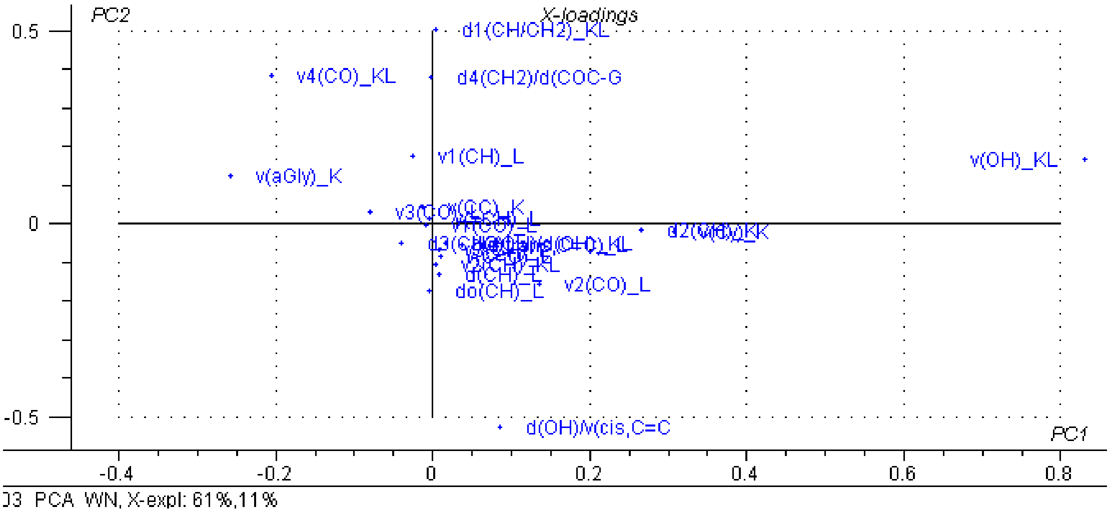

Figure S28. PC2 versus PC1 loadings plot from the PCA analysis of the FTIR band wavenumber data of the β-cyclodextrin/unprocessed and processed chicken breast and thigh lipid complexes, β-cyclodextrin and non-complexed lipid fractions; variable codes contain “v” or “d” for stretching and bending vibrations of the corresponding bonds (in parenthesis), followed by the source (“K” for β-cyclodextrin and “L” for lipids); e.g., “v4(CO)_KL” stands for the variable corresponding to the wavenumber of the 4th stretching vibration of the C-O groups at ~1025 cm^{-1} in both β-cyclodextrin and lipids

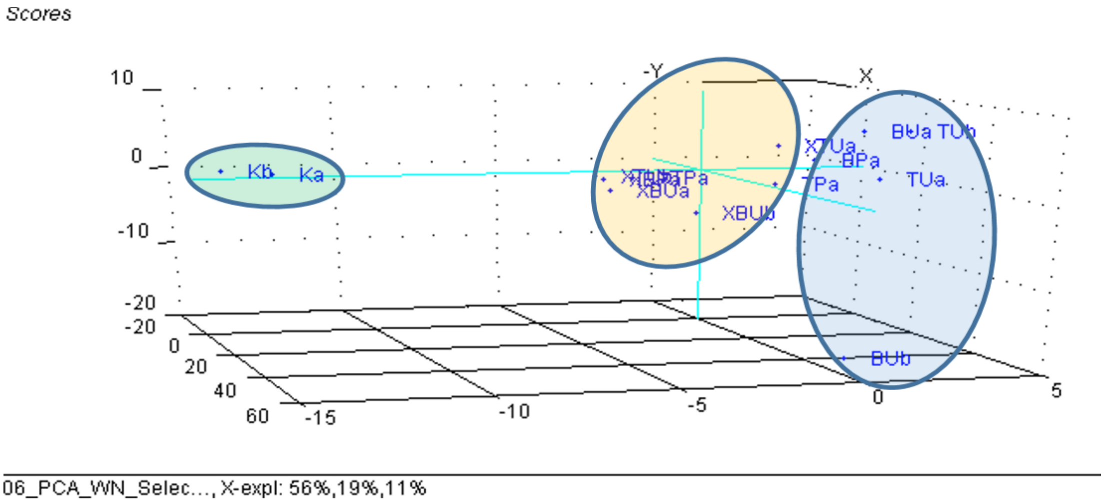

Figure S29. PC3 versus PC1, PC2 scores plot from the PCA analysis of the selected FTIR band wavenumber data (see the text from the article) of the β-cyclodextrin/unprocessed and processed chicken breast and thigh lipid complexes (codes “XBU”, “XBP”, “XTU” and “XTP”), in comparison with β-cyclodextrin (code “K”) and non-complexed lipid fractions (codes “BU”, “BP”, “TU” and “TP”); “a” and “b” stand for duplicate samples

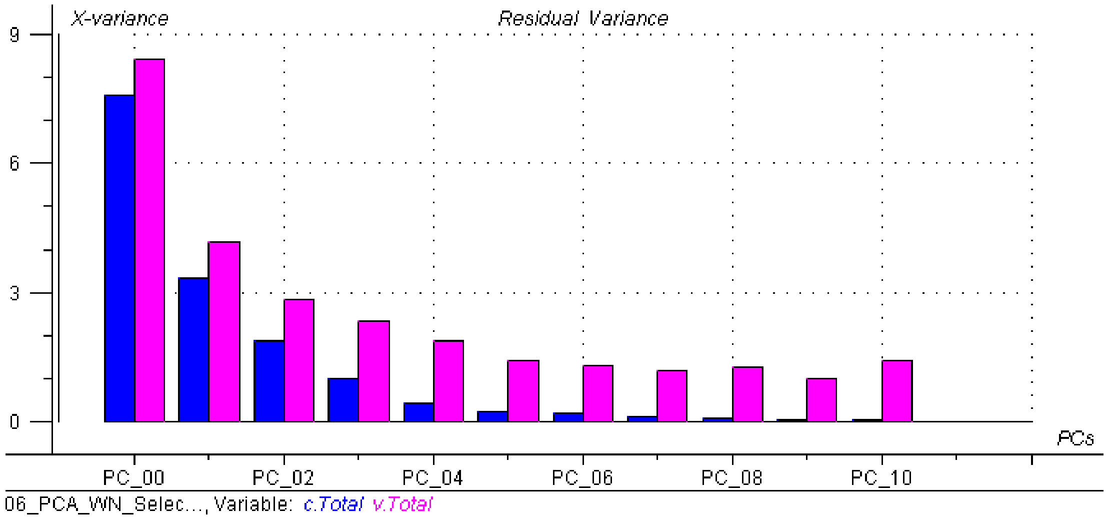

Figure S30. Residual variance from the PCA analysis of the selected FTIR band wavenumber data (see the text from the article) of the β-cyclodextrin/unprocessed and processed chicken breast and thigh lipid complexes, β-cyclodextrin and non-complexed lipid fractions
